# Stronger responses of soil protistan communities to legacy mercury pollution than bacterial and fungal communities in agricultural systems

**DOI:** 10.1038/s43705-022-00156-x

**Published:** 2022-08-09

**Authors:** Shuai Du, Xin-Qi Li, Xiuli Hao, Hang-Wei Hu, Jiao Feng, Qiaoyun Huang, Yu-Rong Liu

**Affiliations:** 1 State Key Laboratory of Agricultural Microbiology, Huazhong Agricultural University, Wuhan, 430070 China; 2 College of Resources and Environment, Huazhong Agricultural University, Wuhan, 430070 China; 3 School of Agriculture and Food, Faculty of Veterinary and Agricultural Sciences, The University of Melbourne, Parkville, VIC 3010 Australia; 4 Hubei Key Laboratory of Soil Environment and Pollution Remediation, Huazhong Agricultural University, Wuhan, 430070 China

**Keywords:** Biodiversity, Microbial ecology, Microbial ecology, Biodiversity

## Abstract

Soil pollution is an important stressor affecting biodiversity and ecosystem functioning. However, we lack a holistic understanding of how soil microbial communities respond to heavy metal pollution in agricultural ecosystems. Here, we explored the distribution patterns and inter-kingdom interactions of entire soil microbiome (including bacteria, fungi, and protists) in 47 paired paddy and upland fields along a gradient of legacy mercury (Hg) pollution. We found that the richness and composition of protistan community had stronger responses to Hg pollution than those of bacterial and fungal communities in both paddy and upland soils. Mercury polluted soils harbored less protistan phototrophs but more protistan consumers. We further revealed that long-term Hg pollution greatly increased network complexity of protistan community than that of bacterial and fungal communities, as well as intensified the interactions between protists and the other microorganisms. Moreover, our results consistently indicated that protistan communities had stronger responses to long-term Hg pollution than bacterial and fungal communities in agricultural soils based on structural equation models and random forest analyses. Our study highlights that soil protists can be used as bioindicators of Hg pollution, with important implications for the assessment of contaminated farmlands and the sustainable management of agricultural ecosystems.

## Introduction

Soil microbiome with vast biodiversity contributes to multiple ecological functions such as climate regulation, nutrient cycling, pathogen control, and pollution remediation in diverse ecosystems [1–4]. Meanwhile, soil microbiome typically responds to environmental disturbance with sensitivity or tolerance to the stresses. For example, environmental pollution caused by human disturbance such as mining have altered core bacterial functions in nutrient and metal cycling reactions [5]. Understanding of microbial stress responses is imperative to evaluate the ecological consequences of environmental pollution. As one of the most prevalent environmental pollutants, some heavy metals such as Cd and Pb have exerted adverse effects on soil microbiome and human health [6, 7]. Among these heavy metals, mercury (Hg) pollution has caused global concerns due to its toxicity and transport over long distances [8]. However, the responses of entire soil microbiomes (including bacteria, fungi, and protists) to chronic Hg pollution remain unclear. Such knowledge is critical to understand the ecological consequences of soil heavy metal pollution.

Soil is the largest reservoir of Hg with a storage of 250~1000 Gg [9, 10], and the ecological effects of soil Hg pollution has become increasingly concerned [11]. Previous studies have advanced our understanding of the impacts of Hg pollution on the diversity and function of bacterial and fungal communities [12, 13]. For instance, Hg pollution reduced bacterial abundance, and altered the proportions of functional genes associated with element cycles and metal transformations [14]. Specifically, elevated soil Hg suppressed the relative abundance of Nitrospirae which was a key group involved in nitrifying processes [14, 15], but facilitated fast-growing opportunistic Firmicutes, Bacteroidetes and Gammaproteobacteria by taking over niches of other bacteria [14, 16]. Moreover, elevated Hg concentration inhibited the growth of soil wood-rotting and ectomycorrhizal (ECM) fungi, which were key groups associated with nutrient cycling [17, 18]. Unfortunately, we lack an understanding of how Hg pollution affects soil protistan community, though other heavy metals such as cadmium (Cd) and lead (Pb) have shown adverse effects on soil protists such as amoebae, diatoms, and flagellates [19, 20]. This lack of knowledge would greatly hamper us to decipher the possible differences in the responses of bacteria, fungi, and protists to Hg pollution.

Different microbial communities could have distinct responses to environmental disturbances [21]. For example, protistan communities were found to be more sensitive to nitrogen fertilization than other microorganisms in agricultural soils [22], while the interaction networks of protistan communities were more vulnerable to warming than that of fungal communities in a coastal soil ecosystem [21]. As a vital component of soil microbiome, protists usually act as the major predators of bacteria, fungi, and other small-size organisms [23], and are considered as the top-down controllers of the community compositions of their prey [24, 25]. Soil protists can also be primary producers, decomposers, and parasites in the belowground micro-food web [26]. For instance, a part of protists are photosynthetic algae and contribute to carbon sequestration in soils [27]. In turn, soil organisms at low trophic levels provide a feedback to their predators via the bottom-up regulation [28]. Environmental changes could alter the multitrophic interactions of microbial communities. For example, warming increased the associations between protists and fungi in the inter-kingdom ecological networks [21]. Despite the pivotal roles of protists in regulating soil microbiomes and sustaining ecosystem functions [29, 30], we still lack a holistic understanding of how long-term Hg pollution affects the soil micro-food webs.

The aim of the present study, therefore, was to explore the diversity patterns and multitrophic interactions of microbial communities in agricultural soils subjected to long-term Hg pollution. We hypothesized that long-term Hg pollution altered the diversity and community composition of soil microbiome with stronger effects on protists than bacteria and fungi, and intensified the multitrophic interactions of microbial communities. To test our hypotheses, we compared the responses of soil protistan, bacterial, and fungal communities at a gradient of legacy Hg pollution in 47 paddy and adjacent upland fields, and identified representative microbial taxa that were strongly associated with Hg pollution. We used a combination of statistical models to identify potential effects of Hg pollution on the attributes of soil microbial communities. This study provides a novel insight into the responses of soil microbiomes to long-term Hg pollution, with implications for sustainable managements of polluted agricultural fields.

## Materials and methods

### Study area, soil sampling and chemical analysis

Soil samples were collected from an over-600-years history of Hg mining region (Fenghuang in Hunan Province and Wanshan in Guizhou Province) in southwest China [31, 32]. Historical discharges from Hg mining operations and ongoing atmospheric deposition led to high Hg concentration in the soils around studied areas. Totally 24 locations were selected along a large gradient of soil total Hg (THg) concentration (ranges from 0.27 to 52.4 mg kg^−1^), as reported previously (Fig. S1) [14]. At each site, three replicated composite soil (0–15 cm depth) were collected from paddy and adjacent upland fields, except an omitted upland site due to the lack of representative field, resulting in a total of 141 composite soil samples in this study. All soil samples were delivered to the laboratory on ice, homogenized, sieved (2.0 mm), and divided into two portions. One portion was stored at 4 °C for soil chemical analysis, and the other portion was stored at −20 °C for the analysis of microbiome.

Soil chemical properties were measured according to the methods described previously [14]. Briefly, soil THg was extracted with HNO_3_-HCl solution and determined via a cold vapor atomic fluorescence spectrometry (CVAFS) [32], methylmercury (MeHg) was extracted with CuSO_4_-methanol solution and determined via an automated MeHg analyzer (TEKRAN 2700 GC-CVAFS) [33]. For other heavy metals (i.e., Cu, Pb, Cd, Zn, Ni, and As), soils were digested by HNO_3_-HF solution and then determined using a 7700X Inductively Coupled Plasma-Mass Spectrometer (Agilent, USA). Soil pH was measured on a fresh soil to water ratio of 1:2.5 using a Delta pH-meter. Total carbon (TC), total nitrogen (TN), and total sulfur (TS) were determined on a LECO TureMac Macro CN analyzer (LECO, St. Joseph, MI, USA). Soil organic carbon (SOC) was measured using the K_2_CrO_7_ oxidation titration method [34]. Soil inorganic nitrogen (NH_4_^+^-N and NO_3_^−^-N) were measured using a SAN + + Continuous Flow Analyser (Skalar, Breda, Netherlands). Dissolved organic carbon (DOC) and total dissolved nitrogen (TDN) were determined with a Shimadzu TOC-TN analyzer (Shimadzu Corp., Kyoto, Japan) [35]. Dissolved organic nitrogen (DON) was calculated as the difference between the TDN reading and the combined NH_4_^+^-N and NO_3_^−^-N reading.

### High-throughput sequencing and bioinformatic analyses

Total genomic DNA was extracted from 0.30 g of soil using the DNeasy PowerSoil Kit (QIAGEN GmbH, Germany) according to the manufacturer’s instructions. Soil DNA quantity and quality were determined using a NanoDrop Spectrophotometer (NanoDrop Technologies Inc., Wilmington, DE, USA). Microbial communities were analyzed using high-throughput sequencing, by targeting the V4 region of 16 S rRNA in bacteria, the internal transcribed spacer (ITS) region for fungi, and the V4 region of 18 S rRNA in protists. Corresponding primer pairs including 338 F/806 R [36], ITS1F/2043 R [37], and TAReuk454FWD1/TAReukREV3 [38] were used, respectively. The purified amplicons were equimolarly mixed and carried out for paired-end sequencing on an Illumina Miseq PE 250 × 2 sequencer (Illumina Inc., San Diego, USA). Bacterial sequences had been used in our previous study [14].

Raw sequences generated from sequencing were processed using Quantitative Insights into Microbial Ecology (QIIME) pipeline (version 1.91) [39]. Briefly, raw reads of each sample were trimmed and merged to paired-end reads, followed by filtering low quality sequences (e.g. chimera) using UPARSE pipeline (version 7.1), and operational taxonomic units (OTUs) were clustered at 97% sequence similarity and a representative sequence of each OTU was selected and used for taxonomic assignments [40]. Bacterial, fungal, and protistan OTUs were taxonomically assigned by blasting against the SILVA database (version 123) [41], the UNITE database [42], and the Protist Ribosomal Reference (PR2) database (version 4.10) [43], respectively. The obtained sequences were rarefied at the minimum number of sequences per sample (bacteria, 29128; fungi, 39748; eukaryotes, 8345) for downstream analysis. Four samples in the paddy soil were discarded for their sparse sequences. To obtain protistan OTU table, the non-protists taxa including Fungi, Metazoa, Rhodophyta, Streptophyta, Opisthokonta, and ambiguous taxa in Eukaryotes were excluded according to a previous study [22]. The taxonomic OTU tables were manually assigned to acquire the putative functional groups of protists, i.e. consumers, parasites, and phototrophs as described previously [44].

### Identification of bioindicators

To identify the bioindicators of soil Hg pollution, all samples were divided into two groups based on THg concentrations (2.0 mg kg^−1^, 2.5 mg kg^−1^, 4.0 mg kg^−1^, and 6.0 mg kg^−1^) corresponding to the value intervals of soil pH (pH ≤ 5.5, 5.5 < pH ≤ 6.5, 6.5 < pH ≤ 7.5, and pH > 7.5), respectively, according to the soil Hg pollution risk values of agricultural land (Table S1, National standards of the People’s Republic of China, GB 15618-2018). If soil THg concentrations exceeded the values, the sample would be classified as high THg group, otherwise low THg group. We compared this criterion with the standards set in the Finnish legislation for contaminated soil [45], which represent a good approximation of the mean values of different national systems in Europe [46, 47]. We found that the threshold, lower and higher guideline values of THg concentrations were 0.5 mg kg^−1^, 2.0 mg kg^−1^, and 5.0 mg kg^−1^ for agricultural land (Table S1) [45], which were comparable to the risk values in GB 15618-2018. Totally, 44 and 28 samples were classified into the low THg group and the high THg group in the paddy soil, and 50 and 19 samples were classified into the low THg group and the high THg group in the upland soil, according to GB 15618-2018 (Table S2). Soils with low THg concentrations were regarded as no polluted, while soils with high THg concentrations were regarded as polluted. Microbial phylotypes (OTUs) appearing in more than one fifth of the samples and their OTUs with relative abundance more than 0.01% were retained. Indicator species analysis was then performed to identify the microbial phylotypes that were significantly associated with the variation of THg concentrations in all soil samples, using the “multipatt” function implemented in the R package “indicspecies” with 999 permutations [48]. According to the positive or negative correlations between microbial phylotypes and THg concentrations (*P* < 0.05), the bioindicators of phylotypes were assigned to two groups (i.e., high and low THg groups), and were identified as Hg-tolerant or Hg-sensitive species, respectively [49].

### Microbial network construction and visualization

Co-occurrence networks were performed to illustrate potential ecological interactions within microbial communities. To compare the variations of protistan, bacterial and fungal interactions in response to Hg pollution, all soil samples were divided into two groups (i.e., low, and high THg groups) as above described. Only bacterial, fungal, and protistan OTUs appearing in more than one fifth of the samples and their OTUs with relative abundance more than 0.01% were retained, respectively [50]. The networks were inferred based on the Spearman’s correlation matrix constructed with the R package “WGCNA” [51]. The cutoff of correlation coefficients was determined through random matrix theory-based methods [52] using molecular ecological network analysis (MENA, http://ieg4.rccc.ou.edu/mena) [53]. The *P* value for each network was adjusted with false discovery rate method and was smaller than 0.01 [54]. All networks were imported into the Gephi platform (version 0.9.2) and visualized by the Frucherman-Reingold algorithms [55]. The topological features such as average degree, clustering coefficient, and network density for each network were calculated using the R package “igraph” [56]. The differences of topological features between low and high THg soils were calculated as follow: (X_high THg_-X_low THg_)/X_low THg_, where X represented each topological feature.

The meta-community co-occurrence networks were further established to decipher the inter-kingdom interactions of protists, bacteria, and fungi for low and high THg soils. The criterion of filtering OTUs was described above. Spearman’s correlations between bacterial, fungal, and protistan communities were calculated via the R package “WGCNA”. The cutoff of correlation coefficients and *P* values for each network were similar as above described. Particularly, three trophic guilds (i.e., consumers, phototrophs, and parasites) of protistan communities were classified to observe their associations with bacterial and fungal phylotypes. The networks were visualized using Cytoscape (version 3.7.2, https://cytoscape.org/). The numbers of nodes and edges, and the links between protists, bacteria, and fungi were calculated for each network.

### Statistical analysis

We analyzed the diversity and community composition of protists, bacteria, and fungi, and compared their responses to soil Hg pollution. The diversity index (observed OTU richness) of bacterial, fungal, and protistan communities was calculated via the “vegan” package [57] in R platform (version 4.0.2), and Pearson’s correlations between the richness of microbial communities and soil THg or MeHg concentrations (logarithm-transformed) were calculated [58]. Then the *R*^2^ values were used to compare the variations of bacterial, fungal, and protistan diversities in response to Hg pollution. The microbial community compositions were calculated by principal coordinate analysis (PCoA) based on the Bray-Curtis dissimilarity matrices between samples using the “vegan” package, and soil THg or MeHg concentrations were standardized ranging from 0 to 1 according to the following formula: STD = (X − X_min_)/(X_max_ − X_min_) [59] and calculated based on Euclidean distances. Then the partial Mantel correlations between microbial community compositions and THg or MeHg concentrations were computed by 999 permutations via the “vegan” package, and the Mantel statistic *r* values were used to compare the variations of bacterial, fungal, and protistan community compositions in response to Hg pollution [60]. We also compared the responses of three trophic guilds (consumers, phototrophs, and parasites) of protists to soil Hg pollution using the above analyses. Particularly, Pearson’s correlations between the relative abundances of representative protistan taxa (standardized by z-transformation with mean = 0 and standard deviation = 1) and soil THg concentrations were calculated [58]. Spearman’s correlation analysis was used to evaluate the correlations between the diversity (richness) and community composition (first axis of PCoA) of bacteria, fungi, and protists with environmental factors using the “psych” package [61]. Random forest analysis was further performed to identify the important environment factors predicting the variations of soil protistan diversity and community composition, via evaluating the increase in the mean square error between observations and predictions, with 999 permutations in the “rfPermute” package [62].

We further conducted structural equation models (SEMs) to identify the associations of legacy Hg pollution with the diversity and community composition of bacteria, fungi, and protists as well as soil chemical properties in the paddy and upland soils, respectively. An *a priori* and theoretical model assumed that (i) legacy Hg pollution directly affected the diversity and community composition of soil bacterial, fungal, and protistan communities, respectively; (ii) legacy Hg indirectly affected microbial communities via altering soil properties; (iii) legacy Hg indirectly affected protistan community via influencing bacterial and fungal communities as a result of prey-predatory interactions between the different microbial groups. The richness (number of OTUs) was used to determine the diversity of bacterial, fungal, and protistan communities. The first axis of PCoA was used to represent the microbial community compositions. All variables were standardized by z-transformation (mean = 0, standard deviation = 1) to improve normality. AMOS 22.0 (SPSS, Chicago, IL, USA) was used for SEM construction and analysis. Maximum likelihood estimation was used to fit the covariance matrix to the model [63]. The parameters including root mean square errors of approximation (RMSEA < 0.08), *χ*^2^ value (*P* > 0.05), goodness-of-fit index (GFI > 0.90) were used to indicate the model fitness [64]. The standardized total effects (STEs) were calculated to quantify the effects of abiotic factors (legacy Hg, soil chemical properties) and biotic factors (bacterial community, fungal community) on the changes of protistan community in agricultural soils.

## Results

### Diversity of soil microbiome along a Hg gradient

**Fig. 1:**
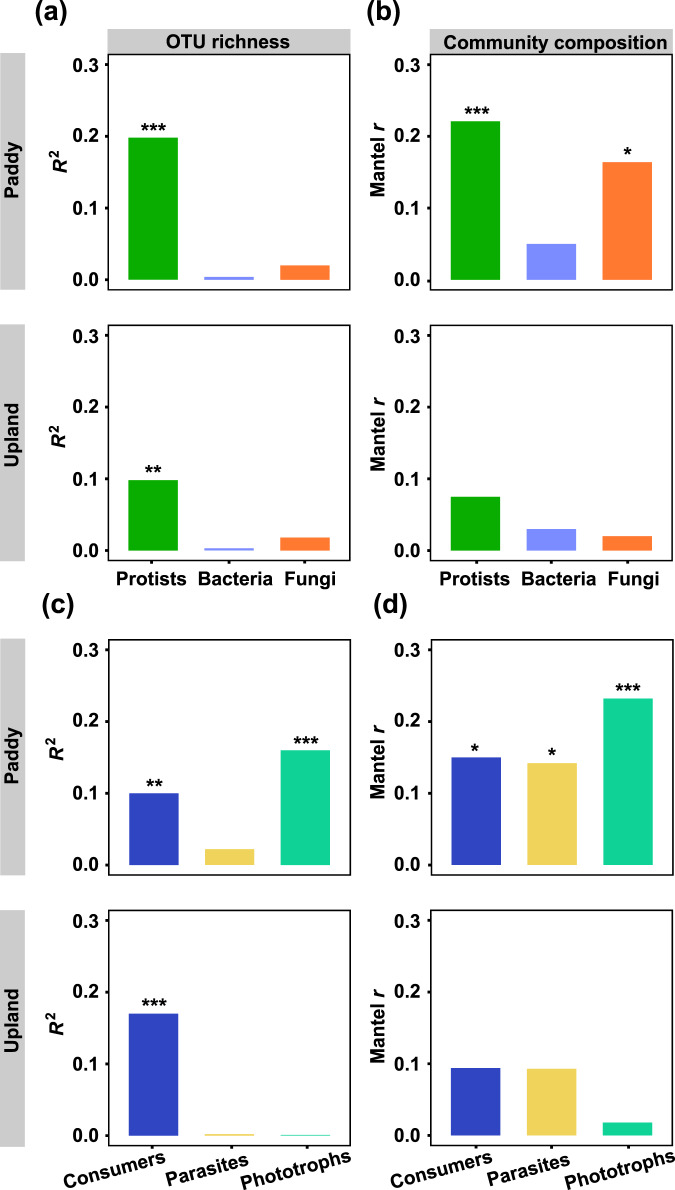
Correlations between the diversity and community composition of protists, bacteria, fungi, and soil THg concentrations. The *R*^2^ of Pearson’ correlations reflect coefficients between the diversity of protists, bacteria, and fungi and soil THg concentrations (**a**). The Mantel statistic *r* reflects correlations between the community composition of protists, bacteria, and fungi and soil THg concentrations (**b**). The *R*^2^ of Pearson’ correlations reflect coefficients between the diversity of protistan trophic guilds and soil THg concentrations (**c**). The Mantel statistic *r* reflects correlations between the community composition of protistan trophic guilds and soil THg concentrations (**d**). The diversity index was calculated by OTU richness. The community composition was calculated by Bray–Curtis dissimilarity matrices. The columns with asterisks represent significant correlations (*P < 0.05, **P < 0.01, ***P < 0.001).

Legacy Hg pollution had a stronger correlation with the diversity of protists (*P* < 0.01) than that of bacteria and fungi (Fig. 1a), though protistan richness had contrast trends along elevated THg concentrations in the paddy and upland soils (Fig. S2). Consistently, the response of protistan community composition to THg concentrations (Mantel *r*, *P* < 0.01) was also stronger than that of bacteria and fungi in the soils (Fig. 1b and Fig. S3). The richness and community composition of protistan phototrophs were more correlated to elevated THg concentrations than those of consumers and parasites in paddy soils, while the greatest correlation was observed between the richness of protistan consumers and THg concentrations in upland soils (Fig. 1c, d and Figs. S4 and S5). Meanwhile, elevated MeHg concentrations decreased protistan richness in the paddy soil (*P* < 0.01, Fig. S6), but increased the dissimilarity of protistan community in both two types of soil (Mantel *r*, *P* < 0.01, Fig. S7). We further profiled the taxonomic composition of protistan community and found that Chlorophyta and Cercozoa accounted for nearly 60% of the entire community, while the trophic composition showed that protistan consumers accounted for up to 57.1%, which was followed by phototrophs accounting for up to 47.0% (Fig. S8). The taxonomic composition of bacterial community was dominated by Proteobacteria and Actinobacteria in the paddy soil and the upland soil, respectively (Fig. S9). The taxonomic composition of fungal community was dominated by Sordariomycetes in both two types of soil (Fig. S10).

### Bioindicators of soil Hg pollution

**Fig. 2:**
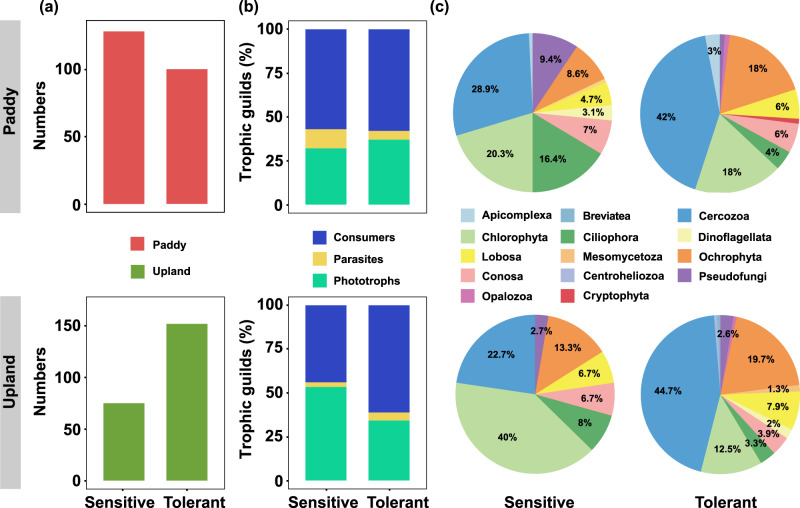
Bioindicators of soil protists identified as Hg-sensitive or Hg-tolerant. Numbers of protistan bioindicators identified as Hg-sensitive or Hg-tolerant (**a**). Proportion of three trophic guilds of protistan bioindicators identified as Hg-sensitive or Hg-tolerant (**b**). The taxonomic compositions (at the phylum level) of protistan bioindicators identified as Hg-sensitive or Hg-tolerant (**c**).

We totally identified 419 protistan bioindicators (including both Hg-sensitive and Hg-tolerant protists) in the soils, accounting for 0.10% in the relative abundance of entire protistan communities, which were much more than bacterial bioindicators (0.05%), and comparable to fungal bioindicators (0.12%). More Hg-sensitive protists were found in paddy soils, while more Hg-tolerant protists were found in upland soils (Fig. 2a). As for the trophic guilds of protists, Hg-tolerant consumers dominated in both types of soil, while Hg-sensitive consumers and phototrophs dominated in the paddy and the upland soil, respectively (Fig. 2b). Specifically, Chlorophyta (phototrophs) was abundant in Hg-sensitive group, while Cercozoa (consumers) was dominant in Hg-tolerant group (Fig. 2c). Additionally, the relative abundance of Ichthyosporea (parasites) and Chrysophyceae (phototrophs) significantly increased along elevated soil Hg concentrations (*P* < 0.05, Fig. S11). We also found that Chloroflexi accounted for larger proportion in Hg-sensitive group than in Hg-tolerant group, while Proteobacteria had opposite results, in bacterial communities (Fig. S12). In parallel, Dothideomycetes accounted for larger proportion in Hg-sensitive group than in Hg-tolerant group, while Eurotiomycetes had opposite results, in fungal communities (Fig. S13).

### Co-occurrence networks of soil microbiome

**Fig. 3:**
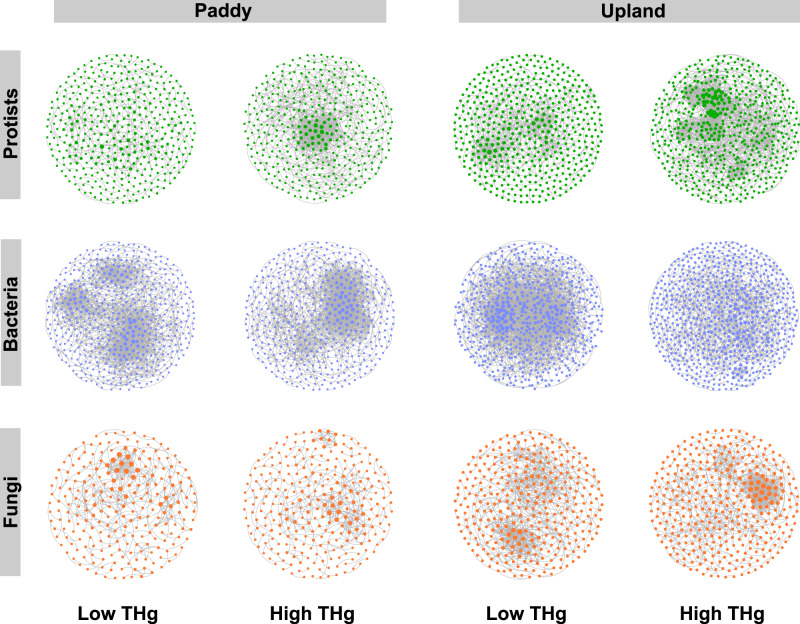
The intra-kingdom networks visualizing Hg pollution effects on the interactions within soil communities. Node colors represent the taxa of protists, bacteria, and fungi in green, blue, and red, respectively. Node size is proportional to the degree, with a larger node having a higher degree.

We assessed the potential associations within protistan, bacterial, and fungal communities in low (i.e., no-polluted) and high THg (i.e., polluted) soils by co-occurrence networks. Our results showed that protistan networks had greater increases of topological indices (including node and edge numbers, average degree, and clustering coefficient, etc.) from no-polluted to polluted soils than bacterial and fungal networks in both paddy and upland soils (Fig. 3), suggesting that the co-occurrence networks of protists are more complexed in polluted soils than in no-polluted soils. For example, the differences of edges between no-polluted and polluted soils were up to 144.69%, 53.13%, and 22.97% (both in the upland soil) for protistan, bacterial, and fungal networks, respectively (Table 1). Consistently, the differences of average degree were up to 109.20%, 55.96%, and 11.49% (both in the upland soil), while the differences of clustering coefficient were up to 114.71%, 20.83%, and 48.72% (both in the upland soil) for protistan, bacterial, and fungal networks, respectively (Table 1).

**Table 1:** Topological indices of networks in Fig. 3.

	Paddy soils						Upland soils					
	Low THg			High THg			Low THg			High THg		
	Protists	Bacteria	Fungi	Protists	Bacteria	Fungi	Protists	Bacteria	Fungi	Protists	Bacteria	Fungi
Number of nodes	402	540	235	398	473	268	476	639	322	556	681	355
Number of edges	399	1772	346	752	1755	371	801	2091	701	1960	980	862
Clustering coefficient	0.38	0.37	0.57	0.51	0.41	0.49	0.34	0.48	0.39	0.73	0.38	0.58
Average degree	1.99	6.56	2.94	3.78	7.42	2.77	3.37	6.54	4.35	7.05	2.88	4.85
Modularity	0.9	0.65	0.83	0.59	0.46	0.85	0.74	0.66	0.72	0.67	0.87	0.71
Density	0.005	0.012	0.01	0.01	0.015	0.01	0.007	0.01	0.01	0.013	0.004	0.01
Positive edges (%)	89.72	78.27	100	85.24	86.72	91.9	89.76	94.55	87.6	93.88	88.78	83.9
Negative edges (%)	10.28	21.73	0	14.76	13.28	8.09	10.24	5.45	12.4	6.12	11.22	16.1

**Fig. 4:**
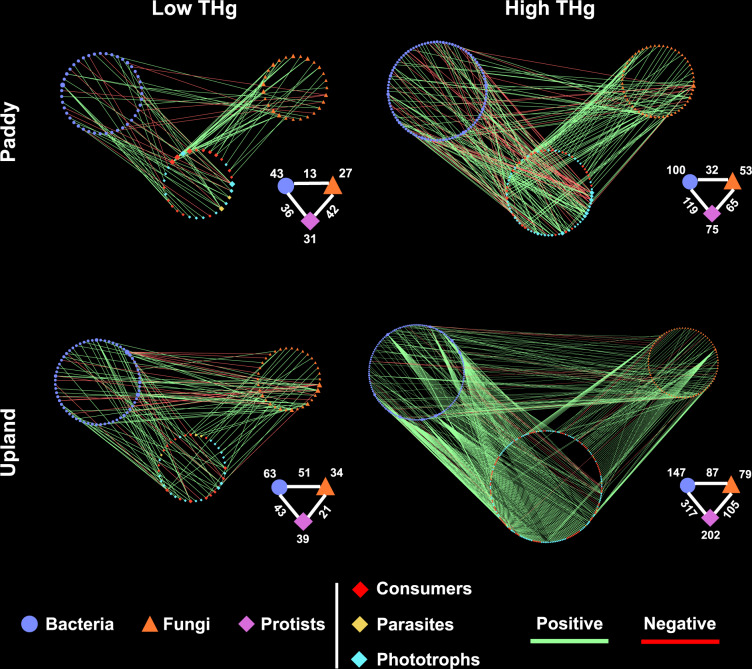
The inter-kingdom networks visualizing Hg pollution effects on the co-occurrence patterns between soil communities. Node shapes (circle, triangle, and lozenge) and colors (blue, orange, and violet) represent microbial groups (bacteria, fungi, and protists), respectively. Node colors of protists (red, cyan, and yellow) represent trophic guilds (consumers, phototrophs, and parasites), respectively. Node size is proportional to the degree, with a larger node having a higher degree. Edge colors represent positive (green) or negative (red) connections between microbial taxa.

To further decipher the potential interactions between microorganisms under legacy Hg pollution, we built the multitrophic networks containing protistan, bacterial, and fungal taxa. The node numbers of protists had greater increase (up to 5.18 times, in the upland soil) than that of bacteria (up to 2.33 times, in the upland soil) and fungi (up to 2.32 times, in the upland soil) in the networks of polluted soils compared to no-polluted soils (Fig. 4 and Table 2). Specially, the node proportion of protistan phototrophs showed larger differences (up to 22.06%, in the paddy soil) between no-polluted and polluted soils than that of consumers (up to 16.89%, in the paddy soil) in the networks (Fig. 4 and Table 2). In addition, we found more interactions between protists and bacteria (up to 317 edges, in the upland soil) than that between protists and fungi (up to 105 edges, in the upland soil) in Hg-polluted soils (Fig. 4 and Table 2). For example, protistan taxa including Sphaeropleales, Chlamydomonadales, Desmodesmus, Staurosira, and Craticula showed more links with bacterial taxa in polluted soils.

**Table 2:** Topological indices of networks in Fig. 4.

	Paddy soils		Upland soils	
	Low THg	High THg	Low THg	High THg
Number of nodes	101	228	136	428
Number of edges	91	216	115	509
Protistan nodes (%)	30.69	32.89	28.68	47.2
Protistan consumer nodes (%)	51.56	34.67	43.59	44.06
Protistan parasite nodes (%)	6.45	1.33	7.69	5.45
Protistan phototroph nodes (%)	41.94	64	48.72	50.5
Bacterial nodes (%)	42.57	43.86	46.32	34.35
Fungal nodes (%)	26.73	23.25	25	18.46
Edges linking protists to bacteria (%)	39.56	55.09	37.39	62.28
Edges linking protists to fungi (%)	46.15	30.09	18.26	20.63
Edges linking bacteria to fungi (%)	14.29	14.81	44.35	17.09
Edges linking consumers to bacteria (%)	6.59	4.63	21.74	24.75
Edges linking consumers to fungi (%)	37.36	16.67	6.96	8.45
Edges linking phototrophs to bacteria (%)	23.08	50.46	14.78	34.97
Edges linking phototrophs to fungi (%)	8.79	12.96	7.83	10.81
Edges linking parasites to bacteria (%)	9.89	0	0.87	2.55
Edges linking parasites to fungi (%)	0	0.46	3.48	1.38

### Associations between Hg pollution and soil microbiome

**Fig. 5:**
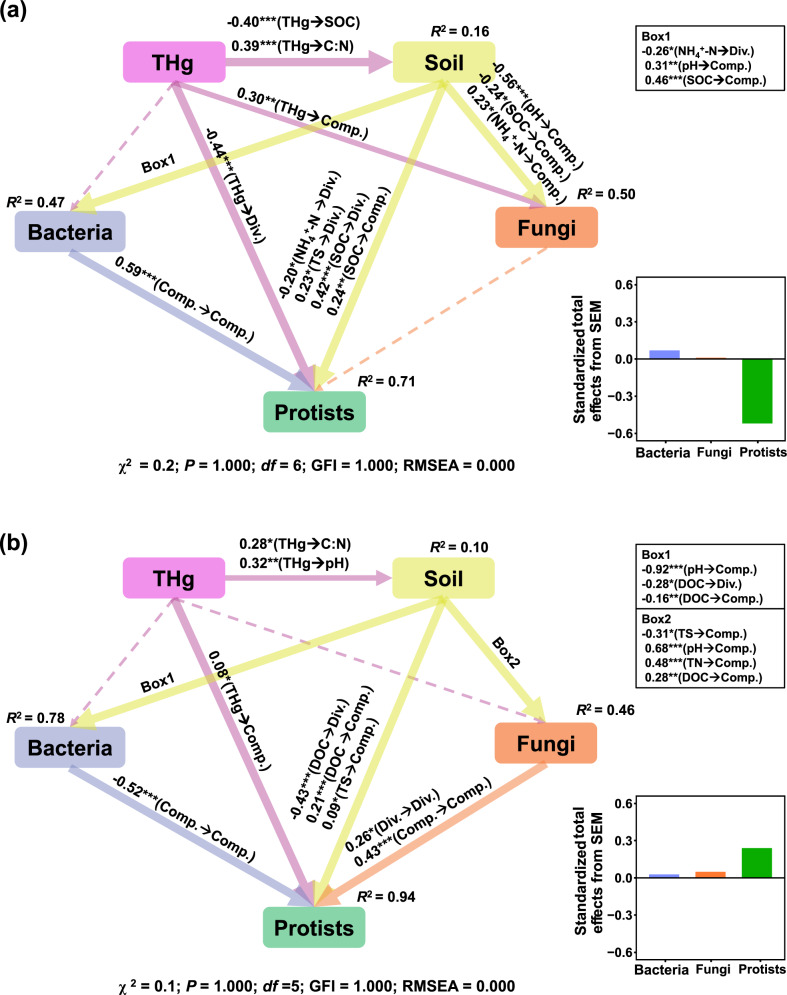
Structural equation models illustrating the associations between Hg pollution and soil microbial communities. The associations were shown in paddy (**a**) and upland (**b**) soils. Continuous and dashed arrows represent the significant and nonsignificant relationships, respectively. Numbers labeled adjacent to the arrow represent the path coefficients, and the width of arrow is in proportion to the degree of path coefficients. *R*^2^ values indicate the proportion of variance explained by each variable. Significance levels are denoted with *P < 0.05, **P < 0.01^,^ and ***P < 0.001. Div. diversity, Comp. community composition, SOC soil organic carbon, DOC dissolved organic carbon, TN total nitrogen, TS total sulfur, C:N carbon nitrogen ratio.

Structural equation models (SEMs) revealed that soil THg concentrations had greater associations (path coefficients) with the diversity of protists than that of bacteria and fungi in both paddy and upland soils (Fig. 5), suggesting that protistan diversity was more responsive to legacy Hg pollution than bacteria and fungi. Our SEMs also showed indirect associations between Hg pollution and protistan community through affecting soil properties such as dissolved organic carbon (DOC) and pH or affecting bacterial community composition in both paddy and upland soils (Fig. 5 and Fig. S14). Importantly, random forest analyses revealed that Hg was the most important predictor explaining the diversity of protists (Fig. S15). Consistently, we found strong Spearman’s correlations between the richness of protistan community and soil THg concentrations, and the composition of protistan community was positively correlated with soil pH, Ni, Cu, and Zn concentrations (Fig. S16).

## Discussion

Soil microbiomes are vital components of belowground trophic networks and exert multiple functional roles in ecosystem processes. Although previous studies have revealed great effects of Hg pollution on soil bacteria and fungi, little is known regarding its effects on soil protists and their interactions with bacteria and fungi. Here, we provided solid evidence that the diversity and composition of soil protistan community showed stronger responses to legacy Hg pollution compared to bacteria and fungi in agricultural ecosystems. Moreover, legacy Hg pollution intensified the interactions between protists and the other trophic groups. These results highlight the importance of soil protistan diversity and inter-kingdom interactions for predicting ecological consequence of heavy metal pollution.

Our results suggest that Hg pollution had a stronger effect on the diversity of protists than that of bacteria and fungi. A possible reason is that protists typically have larger body size and cell surface than bacteria and fungi, and may have higher opportunities to contact with the compounds (e.g., Hg), therefore are more responsive to environmental stresses [23, 65, 66]. This is in line with a recent study revealing that the community assembly of organisms with larger body size (e.g., protists) were more influenced by deterministic processes (usually caused by environmental selections) than smaller ones [67]. In parallel, the community assembly of small size organisms (e.g., bacteria) were mainly driven by stochastic processes (usually caused by species dispersals) [67]. Legacy Hg pollution also resulted in greater changes in the community composition of protists than that of bacteria and fungi, which agrees with a recent study highlighting larger variations of protists than other microorganisms in response to multiple heavy metal contamination (including As, Ni, Pb, Cu and Cd) [68]. In addition, protists may have a narrower habitat niche breadth than bacteria and fungi [69], which could thereby lead to larger variation of the diversity and community composition in response to the pollution stress [70]. The inconsistent responses of protists to Hg pollution in the two types of soil could attribute to the differences in water condition and oxygen availability resulting from distinct management practices [71, 72]. Water-saturated system like paddy field usually has low oxygen availability and could favor the production of MeHg [73] which has a higher toxicity to soil protists, whereas water-unsaturated system like upland field usually has high oxygen availability, facilitating MeHg detoxification [74]. In addition, paddy soil typically has a relatively homogenized environment which supports more opportunity of protists to touch with Hg. Therefore, protists could be more sensitive to Hg pollution in paddy soils compared with those in uplands. Given the fact that most protists are aquatic or semi-aquatic organisms and move along watershed [66, 75], they could be more sensitive to environmental changes in water-saturated systems. Intriguingly, we found consistent increases in protistan community dissimilarity in polluted paddy and upland soils, despite inconsistent changes in protistan diversity in two soils, suggesting that protistan communities could have adapted to long-term environmental pollution through different response modes in diversity in paddy and upland soils [76, 77].

In this study, protistan consumers rather than other trophic guilds were dominant in the soils, which is in line with the distributions of soil protists from a global-scale investigation [44]. A large proportion of protistan phototrophs were sensitive to Hg pollution, suggesting that certain phototrophs could act as bioindicators to assess soil Hg pollution level. Meanwhile, most protistan consumers were Hg-tolerant in the soils, suggesting that consumers might be important to maintain the stability of protistan community in Hg polluted soils. These results are similar to recent studies showing that protistan phototrophs were highly susceptible to livestock manure spiked with antibiotics [78], while the proportion of certain protistan consumers (e.g., Filosa-Sarcomonadea) was increased along antibiotic exposures [79]. It has been proposed that soil protistan consumers can be predicted by biotic factors including invertebrate and bacterial communities, while abiotic factors were the best predictors of phototrophs [80]. The predator-prey relationship between protistan consumers and other microorganisms could respond to environmental stresses, for example, increasing predation to relieve the adverse effects [68]. Meanwhile, protistan phototrophs as primary producers are easily influenced by environmental stresses like organic compound pollution [81]. In addition, we noted the different responses of protistan phototrophs and consumers in paddy and upland soils, suggesting that water management is also important in regulating the effects of Hg pollution on protistan community in agricultural soils.

Furthermore, elevated Hg intensified the interactions within protistan community, and the change of interactions was larger than bacterial and fungal communities. The increased interactions within protistan community could be attributed to the adaption to long-term Hg pollution. For example, chronic environmental perturbation like hydrocarbon contamination maintained the initial microbial community structure [82]. Consistently, soil microbial community gradually recovered after a fumigation in facility agricultural systems [83]. These results and examples collectively suggest that microbial community could recover and be stable after a long-term disturbance through their mutual interactions. The multitrophic interactions among microorganisms are regarded as potential forces driving the distribution of soil microbial communities [80]; however, the interactions in soils subject to heavy metal pollution remain largely unexplored. Our results suggest that protists were the most predominant component in the multitrophic networks and had tight associations with low trophic groups (i.e., bacteria and fungi) in the Hg polluted soils [84]. Similar observation has been reported in a recent study showing that multiple heavy metal contamination (including As, Ni, Pb, Cu and Cd) increased the microbial interactions between protists, bacteria, and fungi [68]. It could be possible that network hubs initially responded to certain environmental stress, and then transferred this response to other components in the network through multitrophic interactions [21]. For example, elevated THg concentrations induced the correlations between protistan consumers and bacteria (e.g., Proteobacteria) in the networks. This finding is supported by a previous study that soil Hg pollution increased the associations between culturable protozoa and bacteria based on a predator-prey relationship [85]. In addition, tight multitrophic interactions promote the resistance of microbial communities towards environmental changes, thus contribute to the stability of microbial functions in agricultural ecosystems [86, 87]. Our SEMs further revealed that long-term Hg pollution displayed strong impacts on the associations between protistan, bacterial, and fungal communities. A recent study also emphasized the role of soil protists on the formation of bacterial community based on a microcosm experiment [77]. Taken together, legacy Hg pollution intensified soil microbiome network associations in agricultural soils.

Our study represents the first assessment of the community diversity and potential multitrophic interactions of the soil microbiome subject to legacy Hg pollution. We provide the multidimensional evidence (diversity, community composition, and biotic interactions) that protistan communities have stronger responses to Hg pollution than the other microorganisms. Moreover, we show that legacy Hg increased the proportion of protists in the trophic networks and intensified their interactions with bacteria and fungi. The sensitive protistan taxa such as Ciliophora and Chlorophyta can be used to fast diagnosis of soil Hg pollution. Our study highlights that soil protists can be used as bioindicators of Hg pollution and have potential roles in regulating multitrophic interactions under environmental pollution. Overall, our work provides holistic insights into the diversity patterns and inter-kingdom interactions of soil microbiome subject to legacy Hg pollution. Given the essential roles of soil microorganisms in driving ecosystem multifunctionality, our results have important implications on the understanding of ecological consequences of environmental pollution.

## Supplementary information

Supporting information

## Data Availability

Bacterial, fungal, and protistan sequences have been submitted to NCBI database with the accession number PRJNA803014, PRJNA803009, and PRJNA803017, respectively.
