# Supplementary material for: Stronger responses of soil protistan communities to legacy mercury pollution than bacterial and fungal communities in agricultural systems
Source: ISME Commun. 2022 Aug 9;2:69. doi: 10.1038/s43705-022-00156-x (PMC9723755; doi:10.1038/s43705-022-00156-x)
Supplement: Supplementary file 1 — Supporting information [file 43705_2022_156_MOESM1_ESM.docx]

***Supporting information for***

**Stronger responses of soil protistan communities to** **legacy mercury pollution than bacterial and fungal communities in agricultural systems**

Shuai Du^1,2,†^, Xin-Qi Li^1,2,†^, Xiuli Hao^1,2^, Hang-Wei Hu^3^, Jiao Feng^1,2^, Qiaoyun Huang^1,2,4^, Yu-Rong Liu^1,2,4,*^

^1^State Key Laboratory of Agricultural Microbiology, Huazhong Agricultural University, Wuhan 430070, China

^2^College of Resources and Environment, Huazhong Agricultural University, Wuhan 430070, China

^3^School of Agriculture and Food, Faculty of Veterinary and Agricultural Sciences, The University of Melbourne, Parkville, Victoria 3010, Australia

^4^Hubei Key Laboratory of Soil Environment and Pollution Remediation, Huazhong Agricultural University, Wuhan 430070, China

^†^**Joint First Authors:** These authors contributed equally to this work.

**^*^Corresponding author:**

Dr. Yu-Rong Liu. College of Resources and Environment, Huazhong Agricultural University, Wuhan, 430070, China. E-mail: yrliu@mail.hzau.edu.cn; Phone: (+86) 27-87286165, Fax: (+86) 27-87286165

**This supplementary file includes 16 figures and 2 tables:**

**Fig. S1 to S16, and Table S1 to S2**


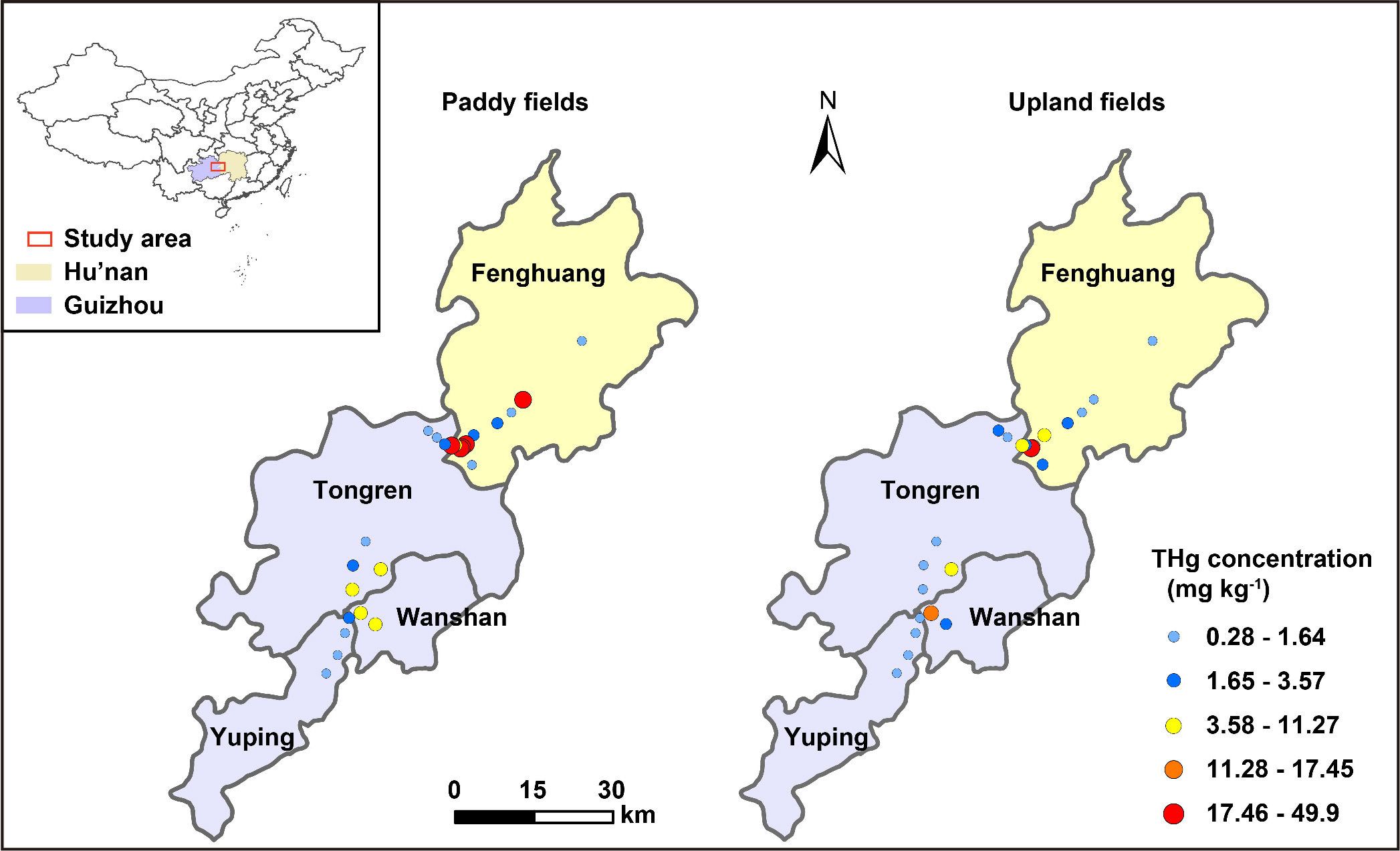


**Fig. S1** Sampling map showing the study area around Fenghuang in Hunan Province and Tongren in Guizhou Province in China. The sizes and colors of nodes in the maps represent soil total Hg (THg) concentrations in paddy and upland fields.


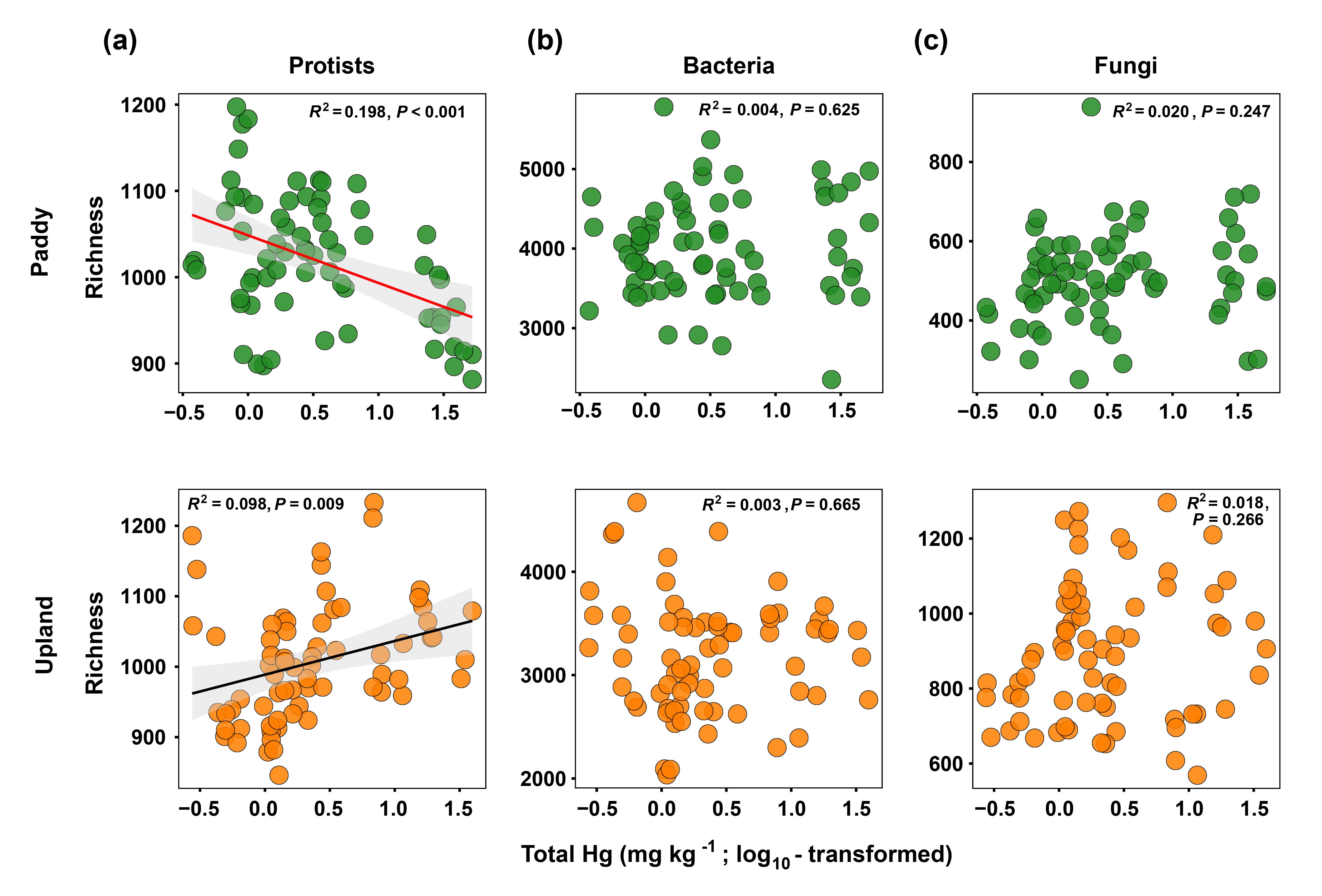


**Fig. S2** Pearson’ correlations between the diversity of protists (a), bacteria (b), fungi (c) and soil total Hg concentrations. The diversity index was calculated by OTU richness. Soil THg concentrations were logarithm-transformed.


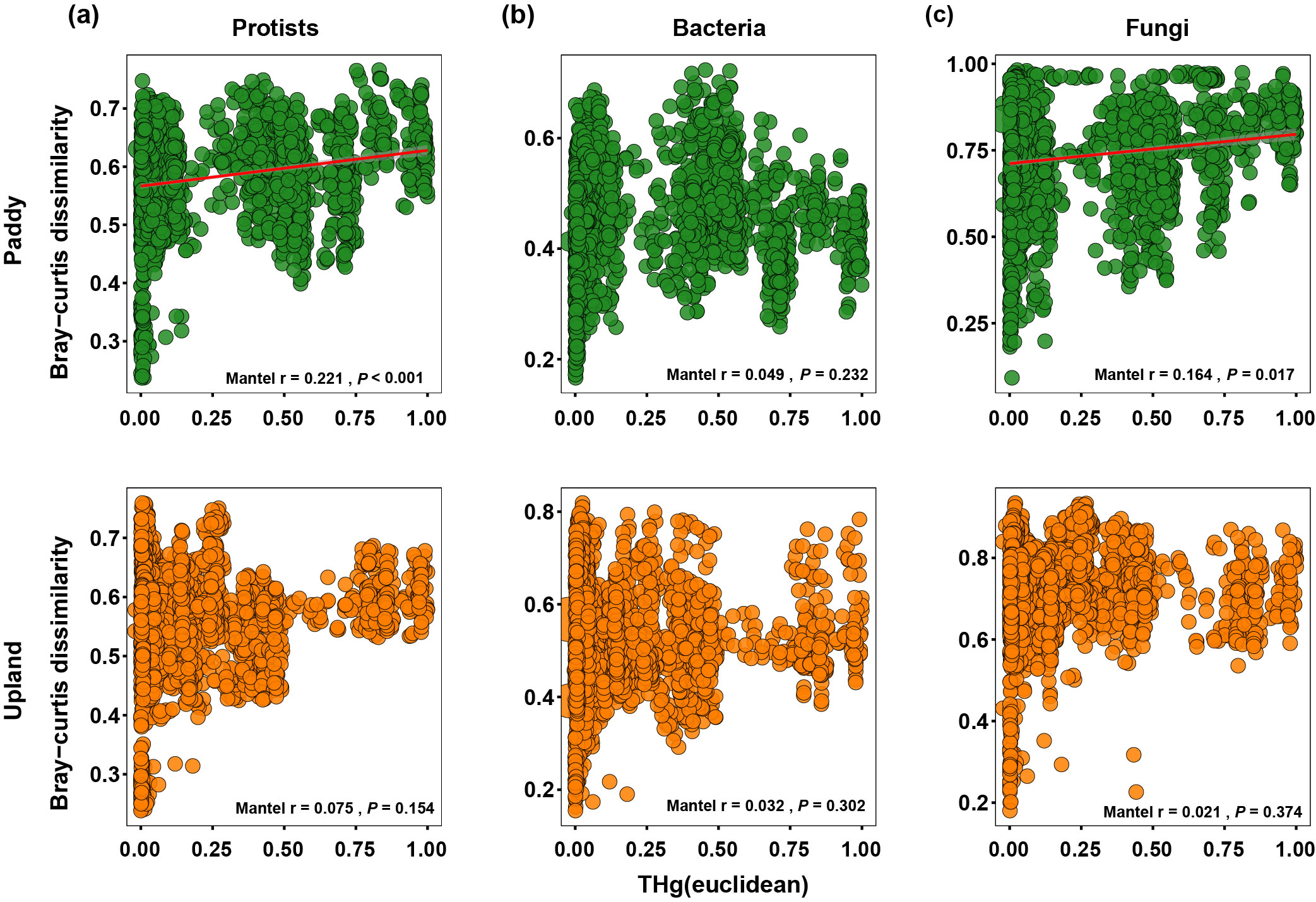


**Fig. S3** Mantel correlations between the community composition of protists (a), bacteria (b), fungi (c) and soil total Hg concentrations. The community composition was calculated by Bray-Curtis dissimilarity matrices. Soil THg concentrations were standardized and Euclidean-transformed.


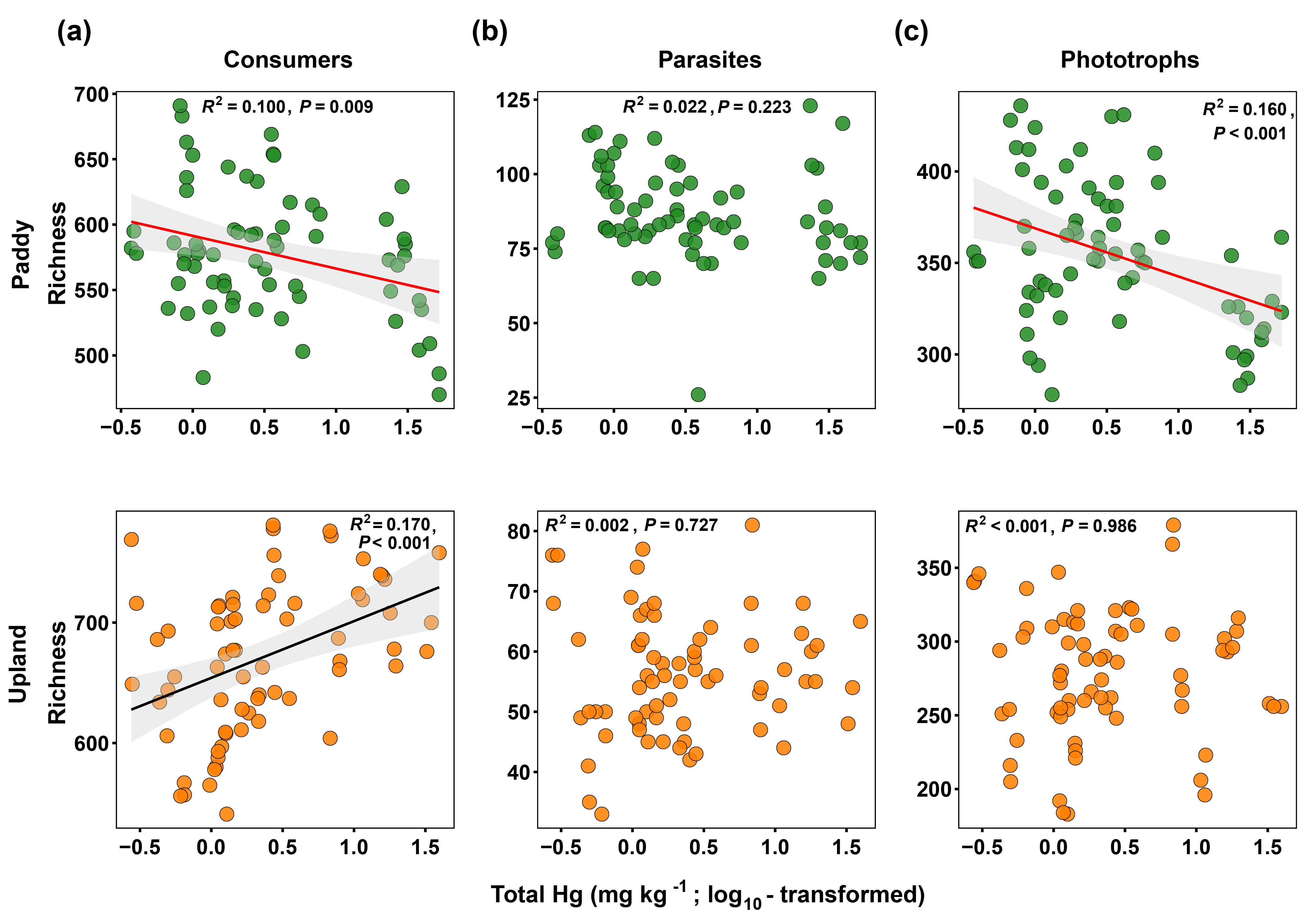


**Fig. S4** Pearson’ correlations between the diversity of protistan trophic guilds i.e., consumers (a), parasites (b), phototrophs (c) and soil total Hg concentrations. The diversity index was calculated by OTU richness. Soil THg concentrations were logarithm-transformed.


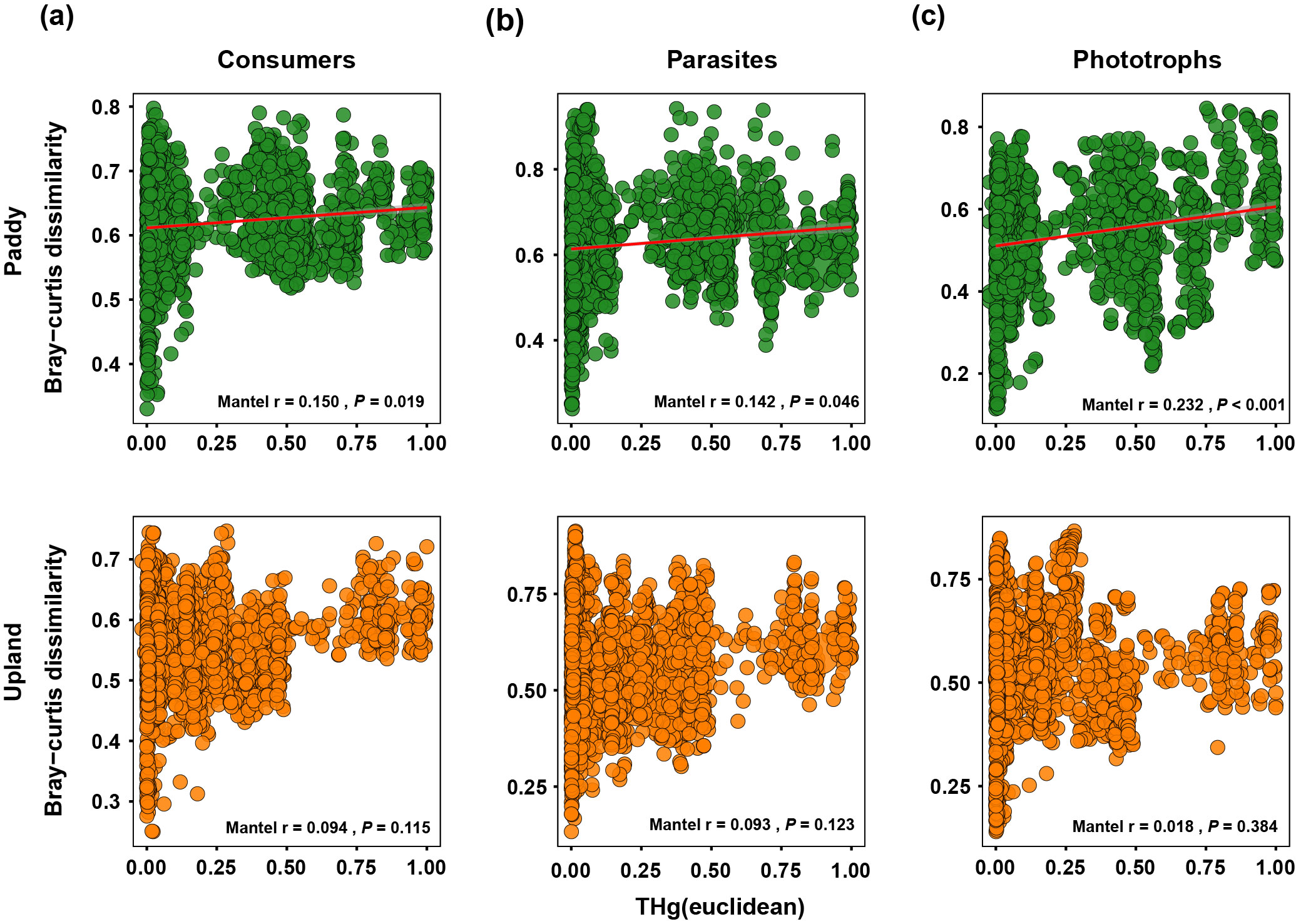


**Fig. S5** Mantel correlations between the community composition of protistan trophic guilds i.e., consumers (a), parasites (b), phototrophs (c) and soil total Hg concentrations. The community composition was calculated by Bray-Curtis dissimilarity matrices. Soil THg concentrations were standardized and Euclidean-transformed.


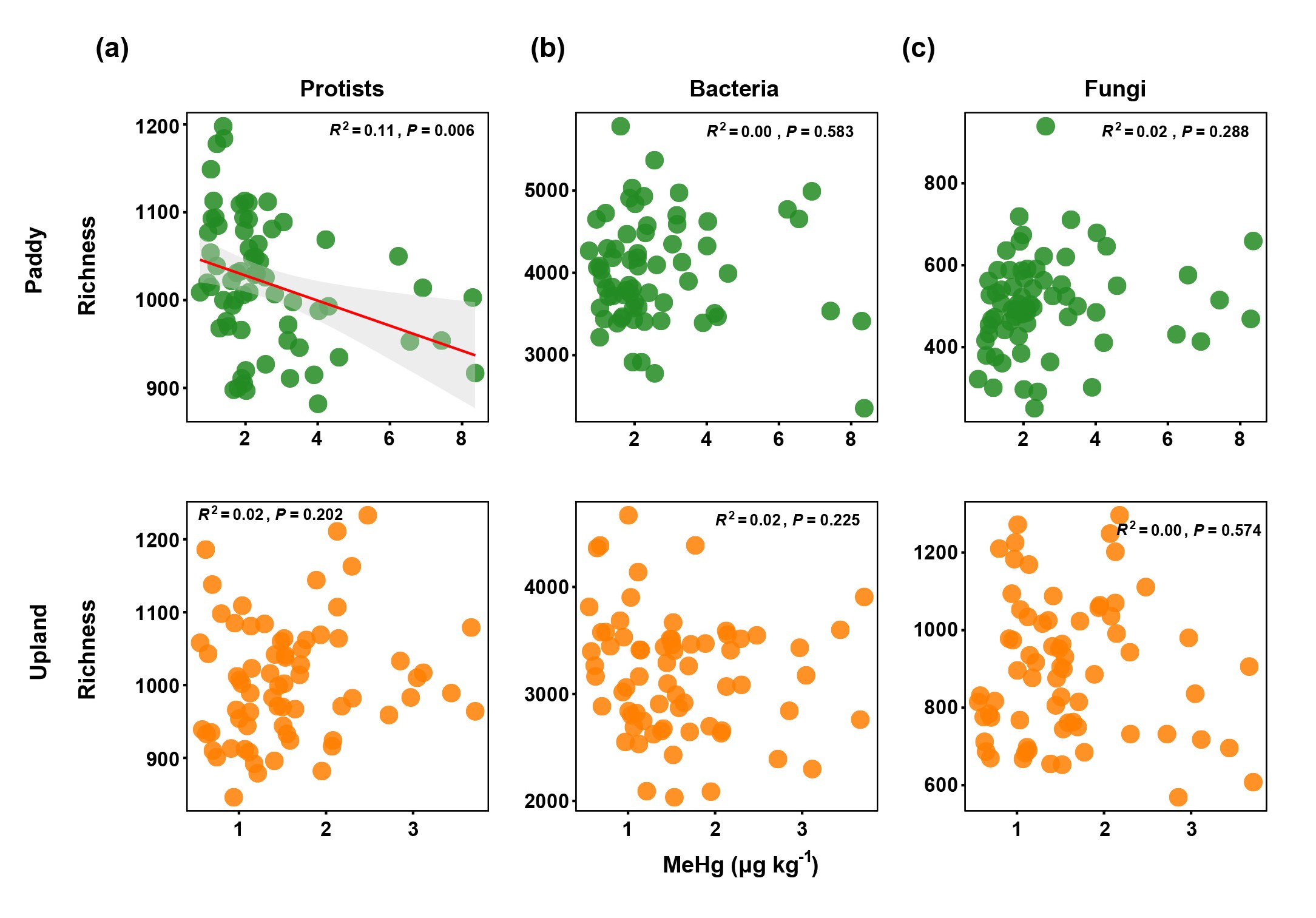


**Fig. S6** Pearson’ correlations between the diversity of protists (a), bacteria (b), fungi (c) and soil MeHg concentrations. The diversity index was calculated by OTU richness.


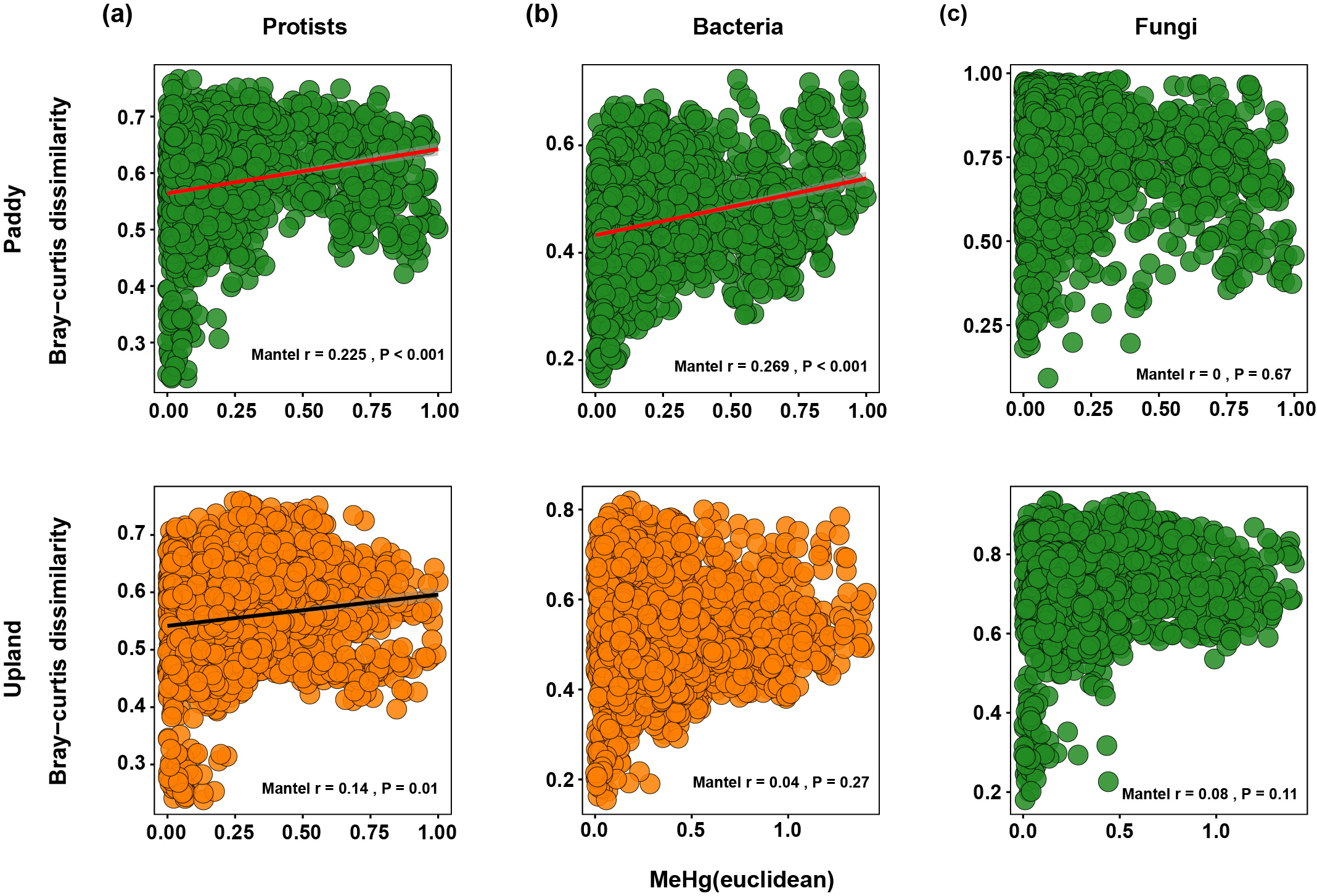


**Fig. S7** Mantel correlations between the community composition of protists (a), bacteria (b), fungi (c) and soil MeHg concentrations. The community composition was calculated by Bray-Curtis dissimilarity matrices.


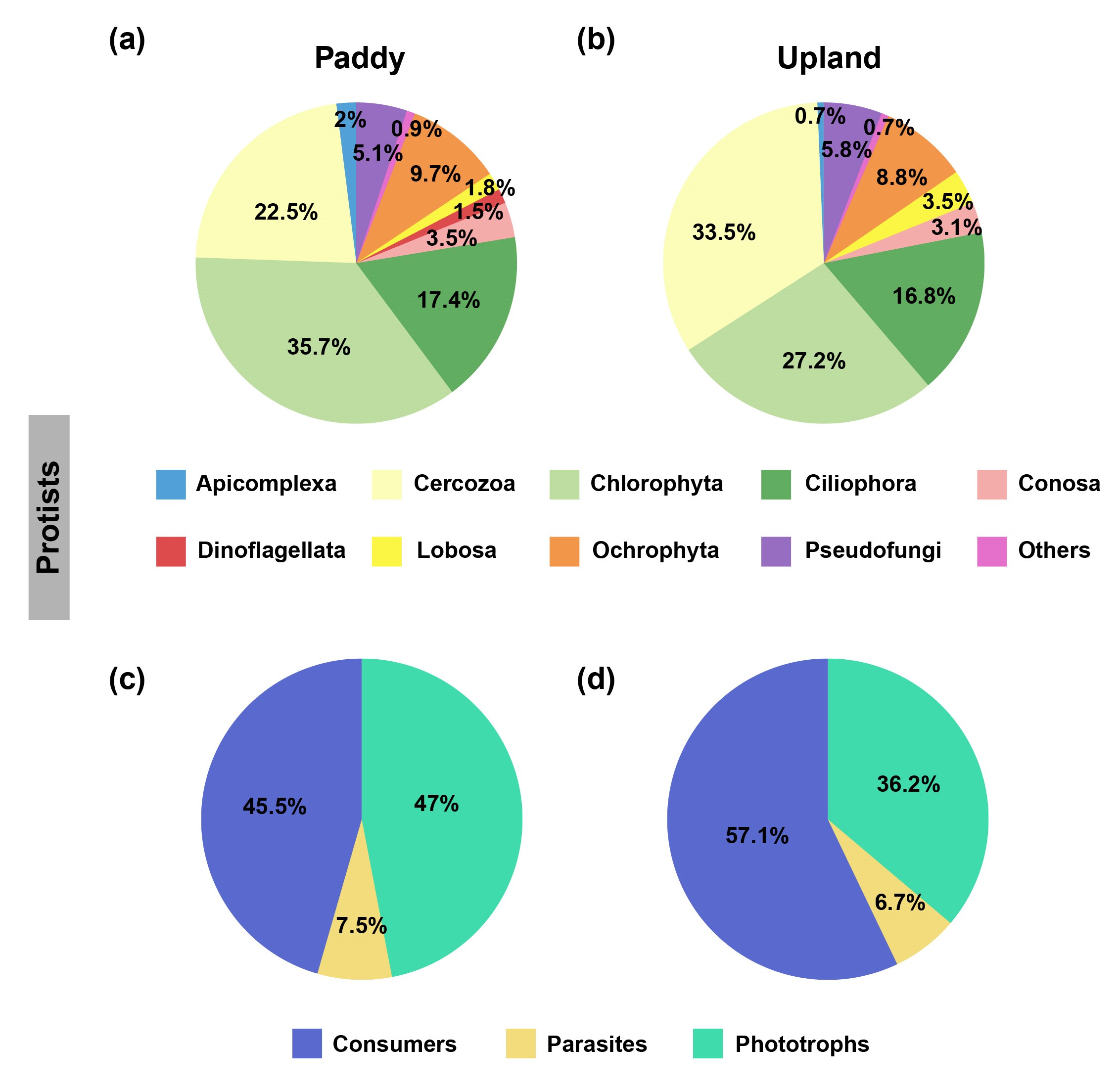


**Fig. S8** The proportions of the relative abundance of protistan taxonomic compositions (at the phylum level) and trophic guilds (consumers, phototrophs, and parasites) in paddy (a and c) and upland soils (b and d).


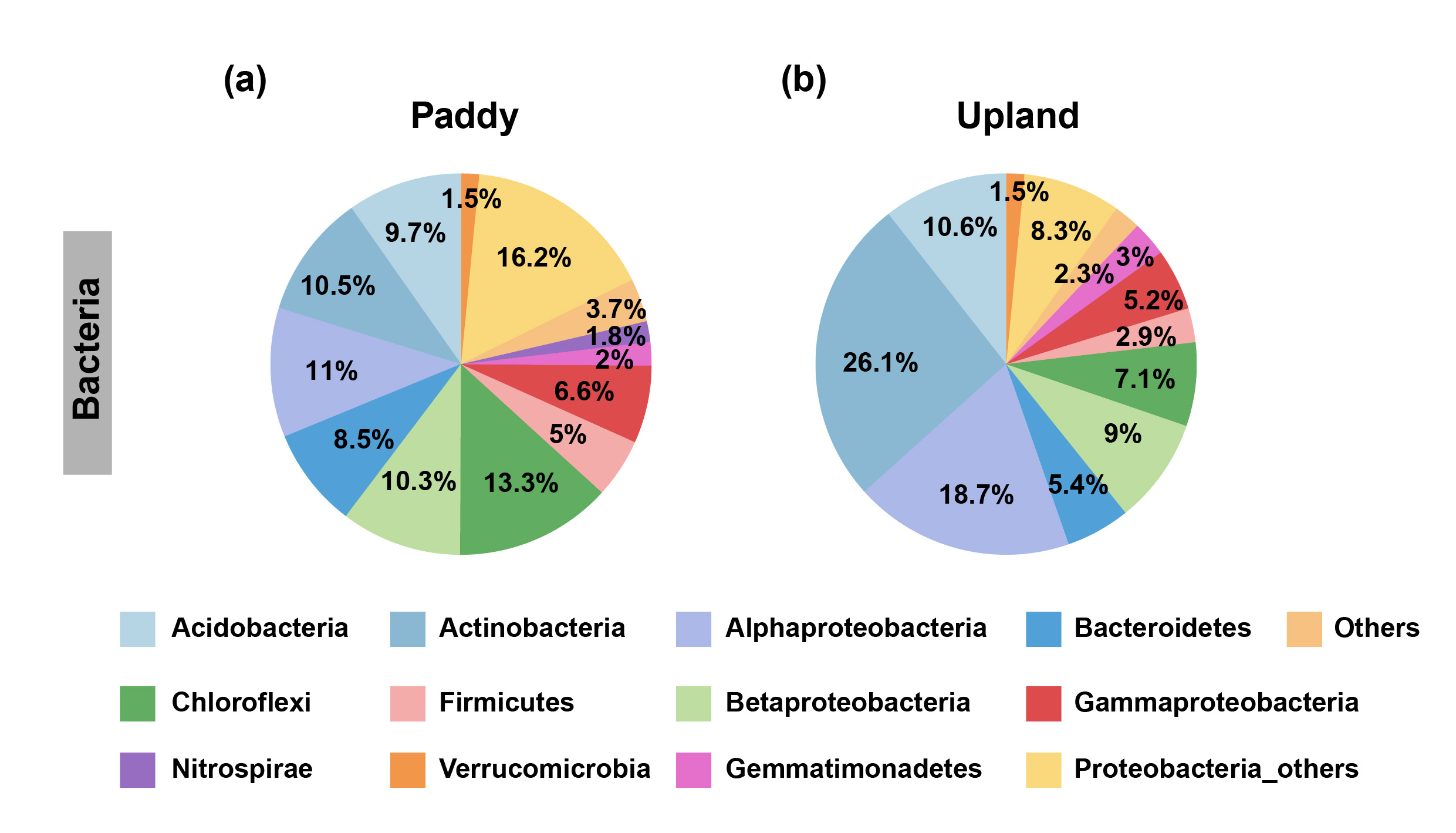


**Fig. S9** The proportions of the relative abundance of bacterial taxonomic compositions (at the phylum level) in paddy (a) and upland soils (b).


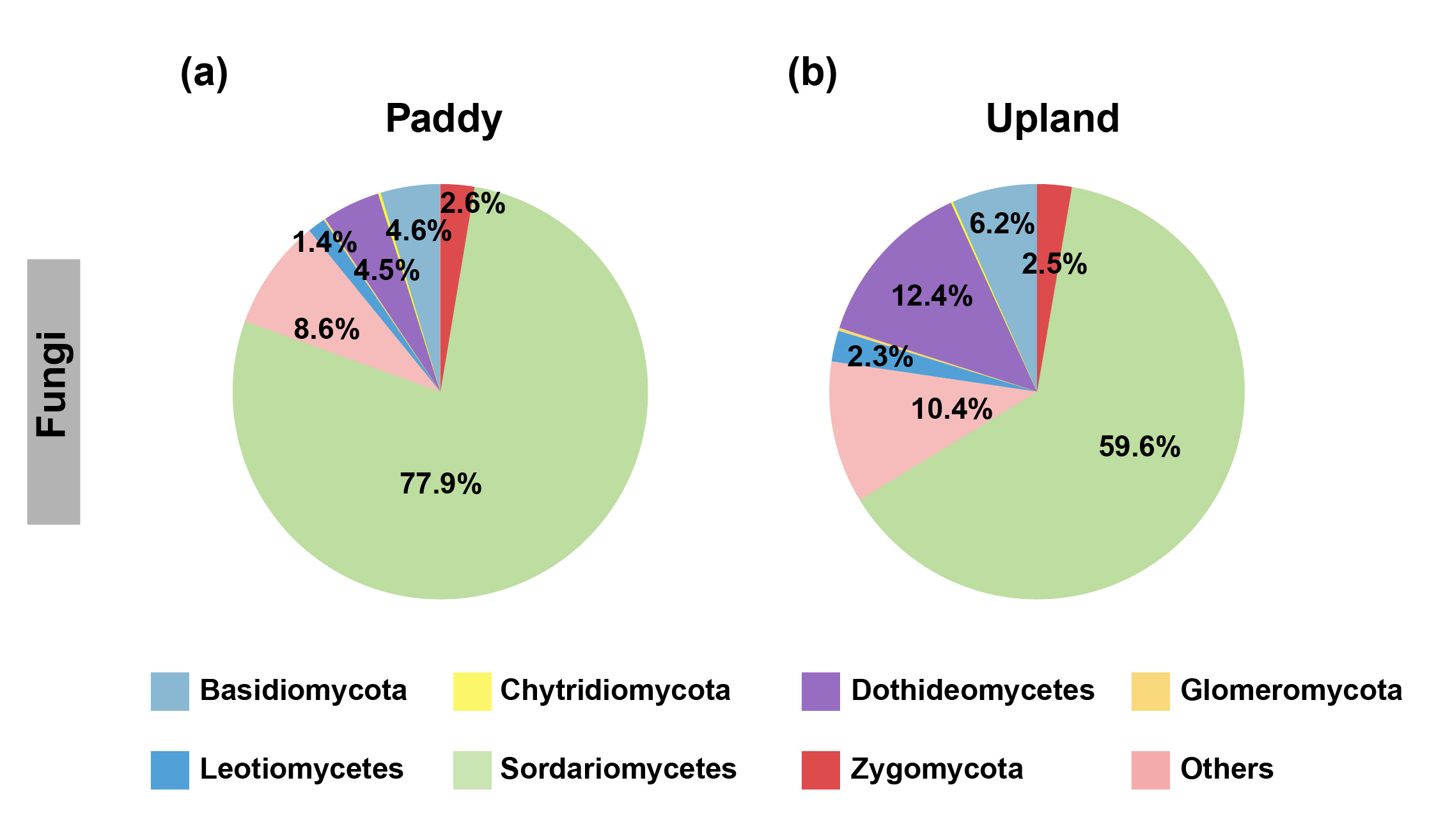


**Fig. S10** The proportions of the relative abundance of fungal taxonomic compositions (at the class level) in paddy (a) and upland soils (b).


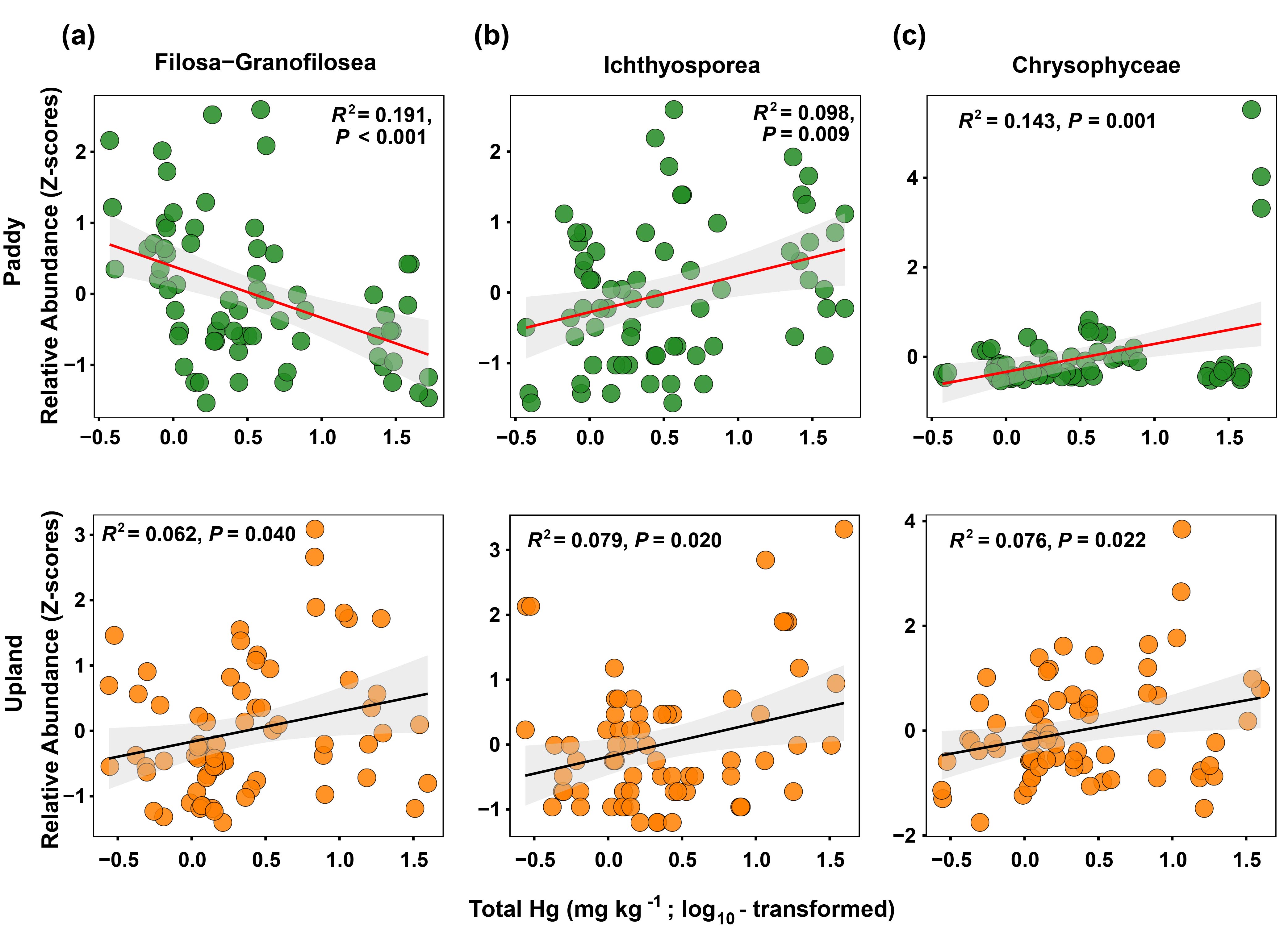


**Fig. S11** Pearson’ correlations between the relative abundance (Z-scores) of protistan taxa (at the order level) and soil total Hg concentrations. Filosa-Granofilosea (a), Ichthyosporea (b), and Chrysophyceae (c) are the representatives of protistan consumes, parasites, and phototrophs, respectively. Soil THg concentrations were logarithm-transformed.


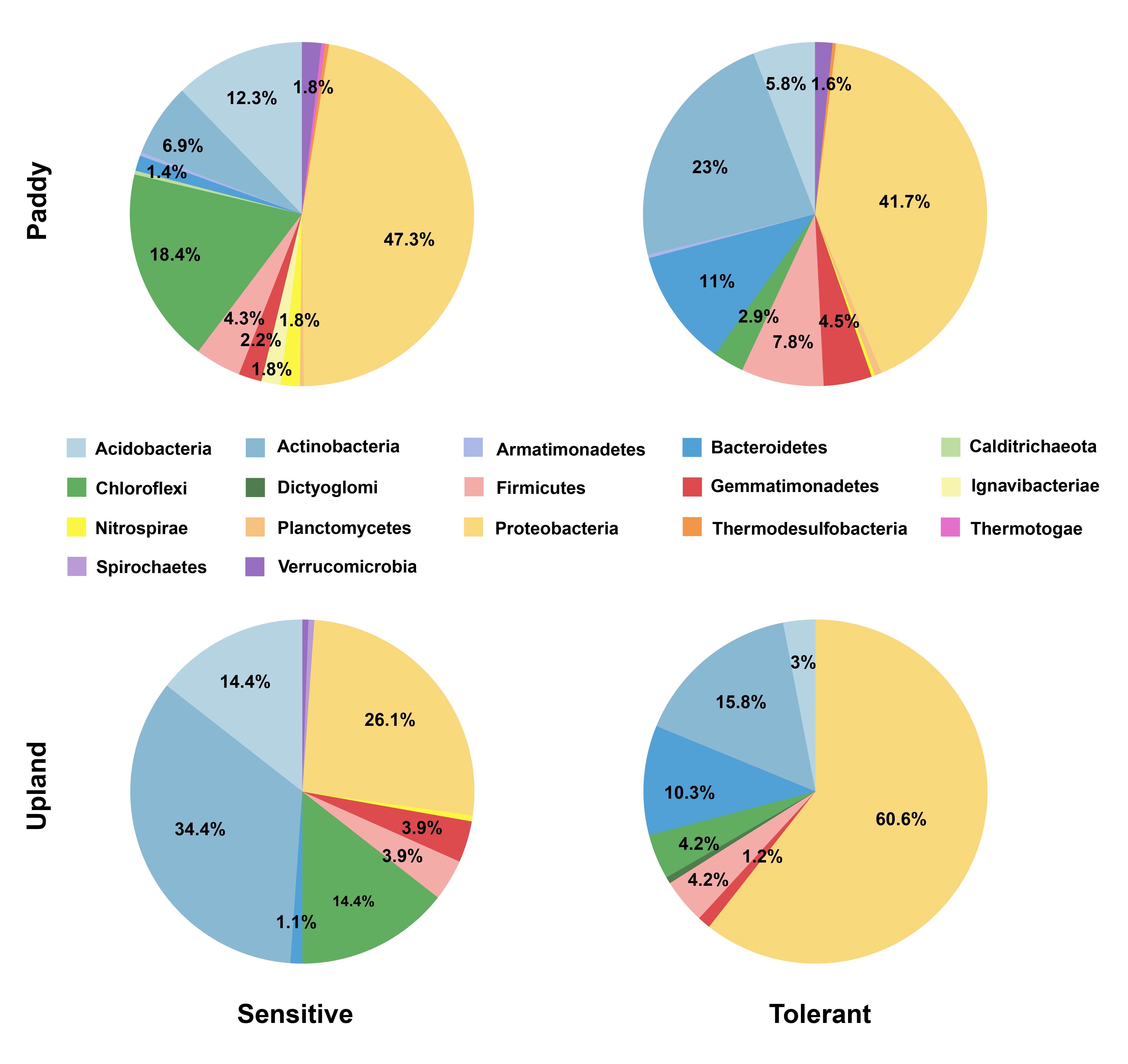


**Fig. S12** The taxonomic compositions (at the phylum level) of bacterial bioindicators identified as Hg-sensitive or Hg-tolerant.


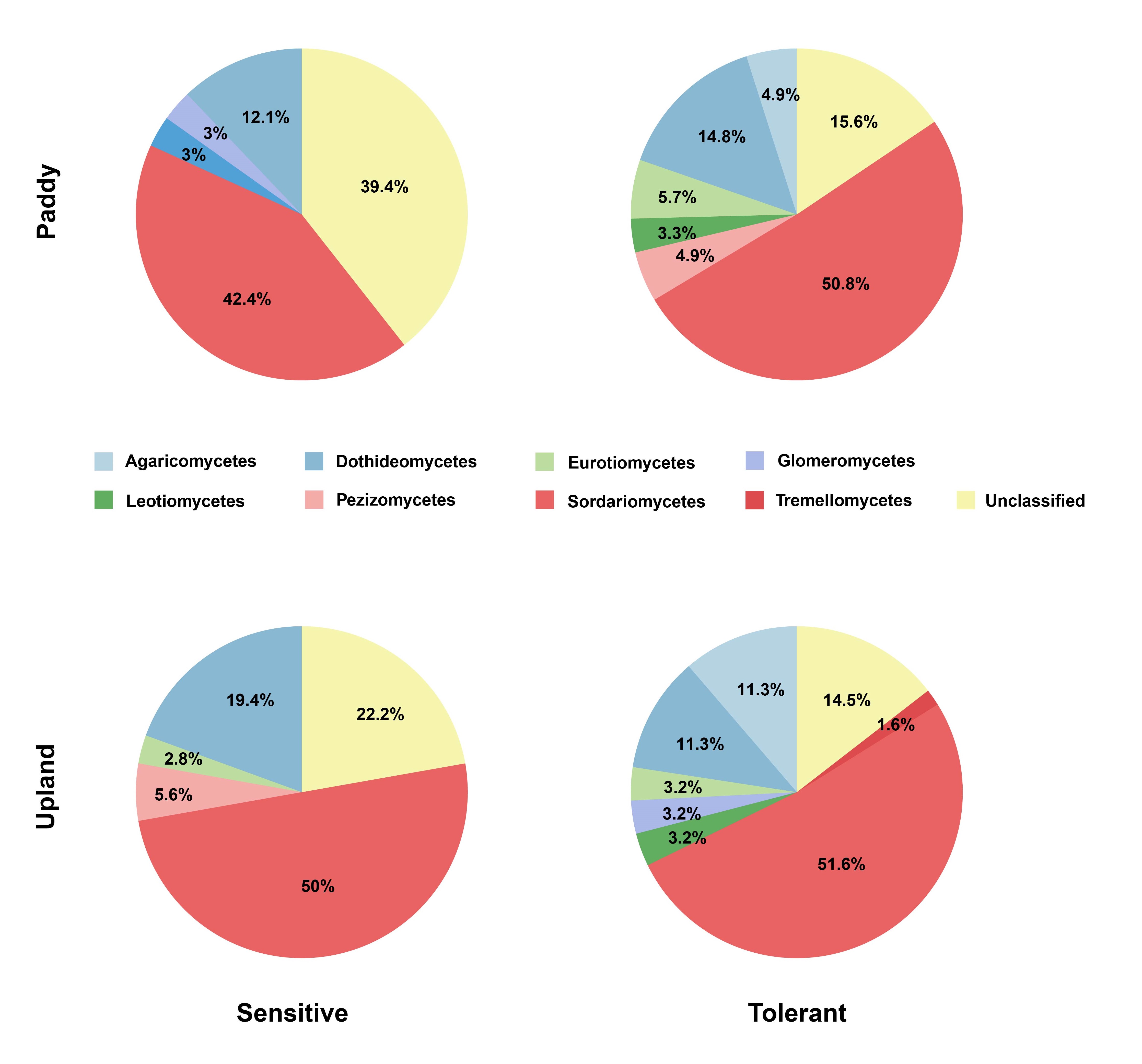


**Fig. S13** The taxonomic compositions (at the class level) of fungal bioindicators identified as Hg-sensitive or Hg-tolerant.


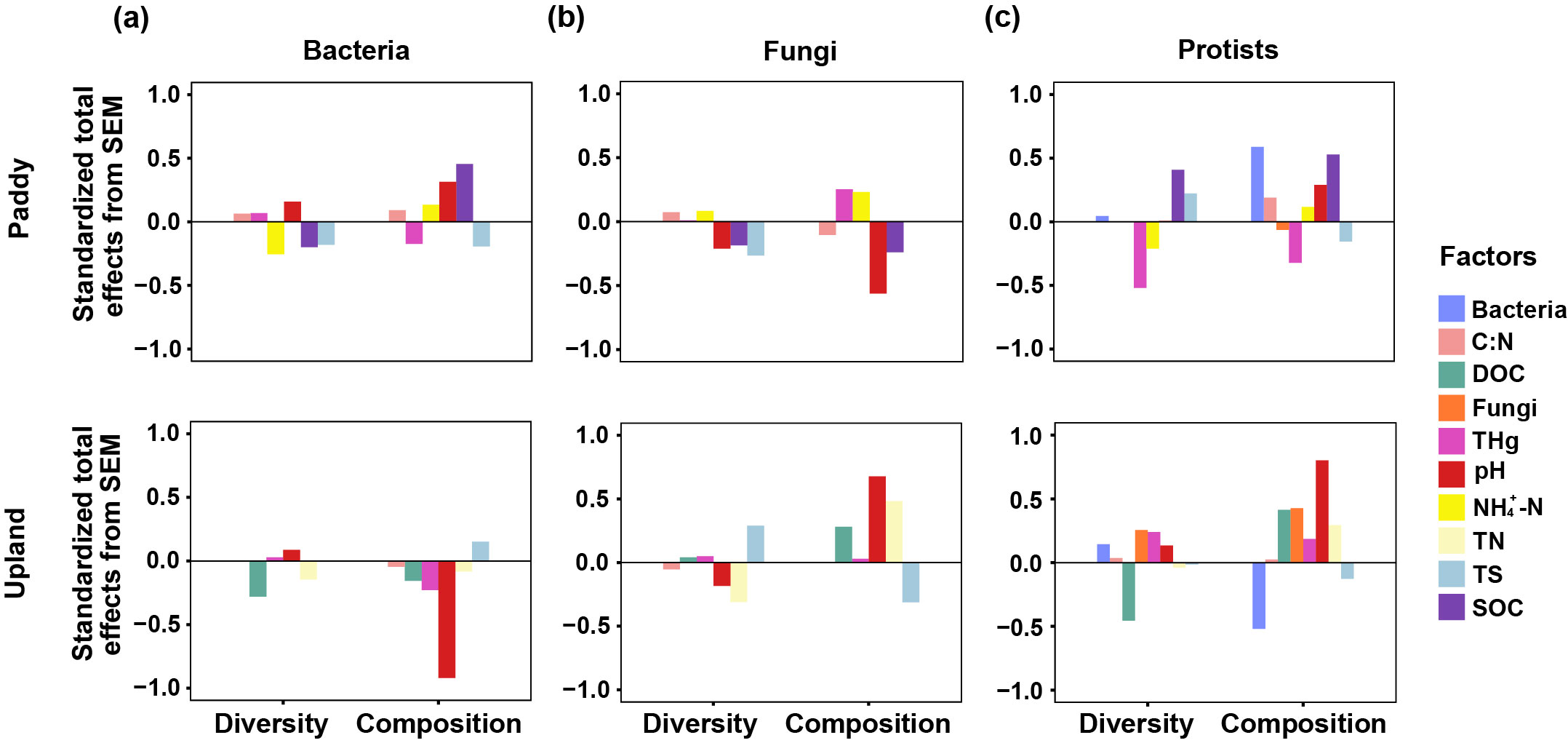


**Fig. S14** Structural equation models showing the standardized total effects of Hg pollution and soil properties on the diversity and community composition of bacteria (a), fungi (b), and protists (c) in paddy and upland soils. Abbreviations: SOC, soil organic carbon; DOC, dissolved organic carbon; TN, total nitrogen; TS, total sulfur; C:N, carbon nitrogen ratio.


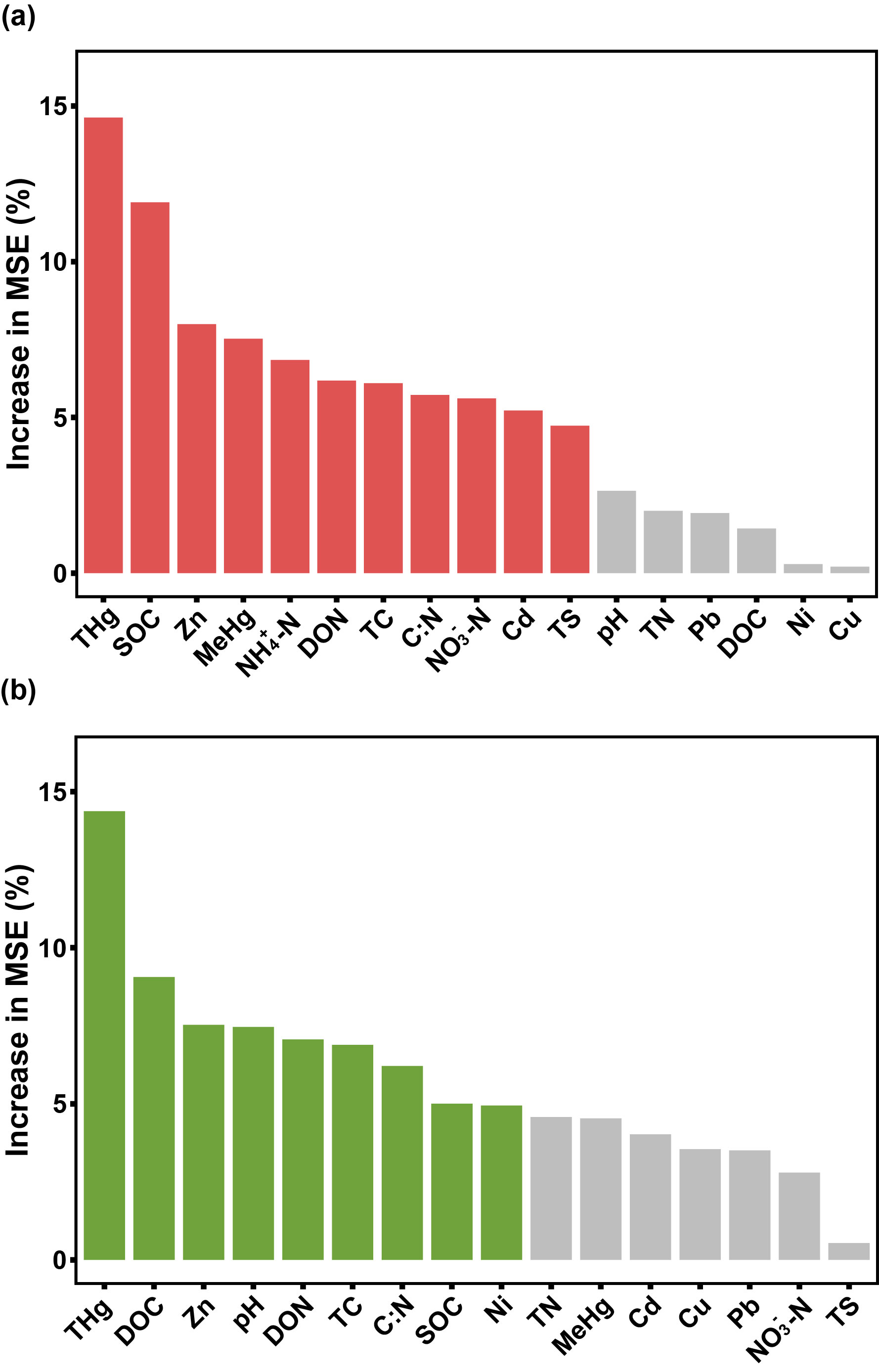


**Fig. S15** Random forest analyses identifying the important predictors of soil properties on the diversity (richness) of protistan communities in paddy (a) and upland soils (b). Significant (*P* < 0.05) predictors of soil properties were colored in red or green, while non-significant (*P* > 0.05) predictors were in gray. Abbreviations: TC, total carbon; TN, total nitrogen; TS, total sulfur; C:N, carbon nitrogen ratio; SOC, soil organic carbon; DOC, dissolved organic carbon; DON, dissolved organic nitrogen.


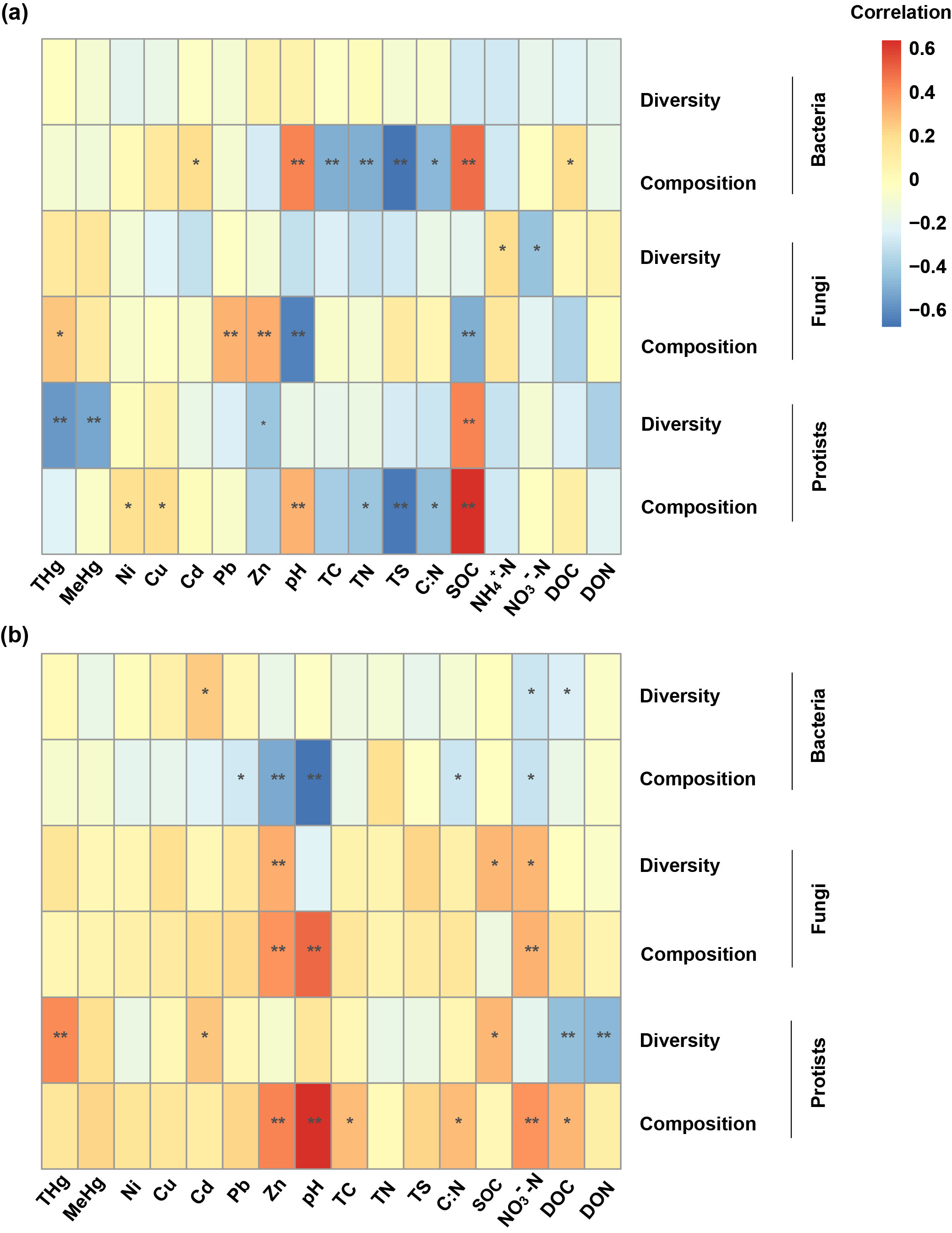


**Fig. S16** Heatmaps showing the Spearman’s correlations between the diversity (richness) and community composition (first axis of PCoA) of bacteria, fungi, and protists in paddy (a) and upland soils (b). The grids with asterisks represent significant difference (^*^*P* < 0.05, ^**^*P* < 0.01, ^***^*P* < 0.001). Abbreviations: TC, total carbon; TN, total nitrogen; TS, total sulfur; C:N, carbon nitrogen ratio; SOC, soil organic carbon; DOC, dissolved organic carbon; DON, dissolved organic nitrogen.

**Table S1** Risk values for soil THg concentration of agricultural land (extract from GB 15618-2018 and MEF-2007).

| GB 15618-2018 | 2.0 mg kg^-1^ (pH ≤ 5.5) | 2.5 mg kg^-1^ (5.5 < pH ≤ 6.5) | 4.0 mg kg^-1^ (6.5 < pH ≤ 7.5) | 6.0 mg kg^-1^ (pH > 7.5) |
| --- | --- | --- | --- | --- |
| MEF-2007 | 0.5 mg kg^-1^ (Threshold value) | 2.0 mg kg^-1^ (Lower guideline value) | 5.0 mg kg^-1^ (Higher guideline value) |  |

**Table S2** The two groups of all samples according to the risk values for soil THg concentration of agricultural land (GB 15618-2018).

| Sample ID | Land use | Soil pH | Soil pH interval | THg (mg kg^-1^) | THg (mg kg^-1^) interval | Group |
| --- | --- | --- | --- | --- | --- | --- |
| p1 | Paddy | 7.31 | 6.5 < pH ≤ 7.5 | 0.84 | Hg ≤ 4 | Low THg |
| p2 | Paddy | 7.31 | 6.5 < pH ≤ 7.5 | 0.91 | Hg ≤ 4 | Low THg |
| p3 | Paddy | 7.25 | 6.5 < pH ≤ 7.5 | 0.91 | Hg ≤ 4 | Low THg |
| p7 | Paddy | 6.75 | 6.5 < pH ≤ 7.5 | 0.67 | Hg ≤ 4 | Low THg |
| p8 | Paddy | 6.77 | 6.5 < pH ≤ 7.5 | 0.74 | Hg ≤ 4 | Low THg |
| p9 | Paddy | 6.77 | 6.5 < pH ≤ 7.5 | 0.79 | Hg ≤ 4 | Low THg |
| p10 | Paddy | 7.19 | 6.5 < pH ≤ 7.5 | 2.19 | Hg ≤ 4 | Low THg |
| p11 | Paddy | 7.27 | 6.5 < pH ≤ 7.5 | 1.95 | Hg ≤ 4 | Low THg |
| p12 | Paddy | 7.16 | 6.5 < pH ≤ 7.5 | 1.92 | Hg ≤ 4 | Low THg |
| p13 | Paddy | 6.93 | 6.5 < pH ≤ 7.5 | 2.75 | Hg ≤ 4 | Low THg |
| p14 | Paddy | 6.69 | 6.5 < pH ≤ 7.5 | 2.76 | Hg ≤ 4 | Low THg |
| p15 | Paddy | 6.86 | 6.5 < pH ≤ 7.5 | 3.18 | Hg ≤ 4 | Low THg |
| p19 | Paddy | 7.49 | 6.5 < pH ≤ 7.5 | 0.39 | Hg ≤ 4 | Low THg |
| p20 | Paddy | 7.45 | 6.5 < pH ≤ 7.5 | 0.37 | Hg ≤ 4 | Low THg |
| p21 | Paddy | 7.47 | 6.5 < pH ≤ 7.5 | 0.40 | Hg ≤ 4 | Low THg |
| p34 | Paddy | 6.96 | 6.5 < pH ≤ 7.5 | 2.75 | Hg ≤ 4 | Low THg |
| p35 | Paddy | 6.92 | 6.5 < pH ≤ 7.5 | 2.81 | Hg ≤ 4 | Low THg |
| p36 | Paddy | 6.67 | 6.5 < pH ≤ 7.5 | 2.56 | Hg ≤ 4 | Low THg |
| p37 | Paddy | 6.59 | 6.5 < pH ≤ 7.5 | 1.10 | Hg ≤ 4 | Low THg |
| p38 | Paddy | 6.41 | 5.5 < pH ≤ 6.5 | 1.08 | Hg ≤ 2.5 | Low THg |
| p39 | Paddy | 6.48 | 5.5 < pH ≤ 6.5 | 1.03 | Hg ≤ 2.5 | Low THg |
| p40 | Paddy | 6.68 | 6.5 < pH ≤ 7.5 | 1.05 | Hg ≤ 4 | Low THg |
| p41 | Paddy | 6.63 | 6.5 < pH ≤ 7.5 | 0.88 | Hg ≤ 4 | Low THg |
| p42 | Paddy | 6.41 | 5.5 < pH ≤ 6.5 | 0.87 | Hg ≤ 2.5 | Low THg |
| p43 | Paddy | 6.97 | 6.5 < pH ≤ 7.5 | 0.90 | Hg ≤ 4 | Low THg |
| p44 | Paddy | 7.08 | 6.5 < pH ≤ 7.5 | 1.00 | Hg ≤ 4 | Low THg |
| p45 | Paddy | 6.89 | 6.5 < pH ≤ 7.5 | 0.82 | Hg ≤ 4 | Low THg |
| p46 | Paddy | 6.81 | 6.5 < pH ≤ 7.5 | 1.77 | Hg ≤ 4 | Low THg |
| p47 | Paddy | 6.47 | 5.5 < pH ≤ 6.5 | 1.83 | Hg ≤ 2.5 | Low THg |
| p48 | Paddy | 6.44 | 5.5 < pH ≤ 6.5 | 1.83 | Hg ≤ 2.5 | Low THg |
| p55 | Paddy | 7.16 | 6.5 < pH ≤ 7.5 | 3.42 | Hg ≤ 4 | Low THg |
| p57 | Paddy | 7.24 | 6.5 < pH ≤ 7.5 | 3.69 | Hg ≤ 4 | Low THg |
| p61 | Paddy | 6.97 | 6.5 < pH ≤ 7.5 | 1.89 | Hg ≤ 4 | Low THg |
| p62 | Paddy | 7.11 | 6.5 < pH ≤ 7.5 | 2.38 | Hg ≤ 4 | Low THg |
| p63 | Paddy | 7.17 | 6.5 < pH ≤ 7.5 | 2.07 | Hg ≤ 4 | Low THg |
| p64 | Paddy | 7.26 | 6.5 < pH ≤ 7.5 | 1.27 | Hg ≤ 4 | Low THg |
| p65 | Paddy | 7.33 | 6.5 < pH ≤ 7.5 | 1.65 | Hg ≤ 4 | Low THg |
| p66 | Paddy | 7.23 | 6.5 < pH ≤ 7.5 | 1.39 | Hg ≤ 4 | Low THg |
| p67 | Paddy | 7.22 | 6.5 < pH ≤ 7.5 | 1.31 | Hg ≤ 4 | Low THg |
| p68 | Paddy | 7.22 | 6.5 < pH ≤ 7.5 | 1.18 | Hg ≤ 4 | Low THg |
| p69 | Paddy | 7.23 | 6.5 < pH ≤ 7.5 | 0.92 | Hg ≤ 4 | Low THg |
| p70 | Paddy | 7.09 | 6.5 < pH ≤ 7.5 | 1.39 | Hg ≤ 4 | Low THg |
| p71 | Paddy | 7.07 | 6.5 < pH ≤ 7.5 | 1.50 | Hg ≤ 4 | Low THg |
| p72 | Paddy | 7.1 | 6.5 < pH ≤ 7.5 | 1.67 | Hg ≤ 4 | Low THg |
| u1 | Upland | 7.1 | 6.5 < pH ≤ 7.5 | 0.42 | Hg ≤ 4 | Low THg |
| u2 | Upland | 6.96 | 6.5 < pH ≤ 7.5 | 0.43 | Hg ≤ 4 | Low THg |
| u3 | Upland | 6.91 | 6.5 < pH ≤ 7.5 | 0.49 | Hg ≤ 4 | Low THg |
| u4 | Upland | 7.33 | 6.5 < pH ≤ 7.5 | 0.28 | Hg ≤ 4 | Low THg |
| u5 | Upland | 7.41 | 6.5 < pH ≤ 7.5 | 0.28 | Hg ≤ 4 | Low THg |
| u6 | Upland | 7.29 | 6.5 < pH ≤ 7.5 | 0.30 | Hg ≤ 4 | Low THg |
| u7 | Upland | 5.15 | pH ≤ 5.5 | 0.65 | Hg ≤ 2 | Low THg |
| u8 | Upland | 4.98 | pH ≤ 5.5 | 0.65 | Hg ≤ 2 | Low THg |
| u9 | Upland | 4.97 | pH ≤ 5.5 | 0.61 | Hg ≤ 2 | Low THg |
| u10 | Upland | 7.09 | 6.5 < pH ≤ 7.5 | 2.75 | Hg ≤ 4 | Low THg |
| u11 | Upland | 7.23 | 6.5 < pH ≤ 7.5 | 2.52 | Hg ≤ 4 | Low THg |
| u12 | Upland | 7.32 | 6.5 < pH ≤ 7.5 | 2.31 | Hg ≤ 4 | Low THg |
| u13 | Upland | 7.16 | 6.5 < pH ≤ 7.5 | 3.39 | Hg ≤ 4 | Low THg |
| u14 | Upland | 6.98 | 6.5 < pH ≤ 7.5 | 3.53 | Hg ≤ 4 | Low THg |
| u15 | Upland | 7.11 | 6.5 < pH ≤ 7.5 | 3.85 | Hg ≤ 4 | Low THg |
| u16 | Upland | 6.11 | 5.5 < pH ≤ 6.5 | 2.16 | Hg ≤ 2.5 | Low THg |
| u18 | Upland | 5.95 | 5.5 < pH ≤ 6.5 | 2.28 | Hg ≤ 2.5 | Low THg |
| u25 | Upland | 6.58 | 6.5 < pH ≤ 7.5 | 2.12 | Hg ≤ 4 | Low THg |
| u26 | Upland | 6.16 | 5.5 < pH ≤ 6.5 | 1.83 | Hg ≤ 2.5 | Low THg |
| u27 | Upland | 6.01 | 5.5 < pH ≤ 6.5 | 2.15 | Hg ≤ 2.5 | Low THg |
| u34 | Upland | 7.19 | 6.5 < pH ≤ 7.5 | 0.98 | Hg ≤ 4 | Low THg |
| u35 | Upland | 6.93 | 6.5 < pH ≤ 7.5 | 1.18 | Hg ≤ 4 | Low THg |
| u36 | Upland | 6.92 | 6.5 < pH ≤ 7.5 | 1.08 | Hg ≤ 4 | Low THg |
| u37 | Upland | 7.19 | 6.5 < pH ≤ 7.5 | 1.64 | Hg ≤ 4 | Low THg |
| u38 | Upland | 7.27 | 6.5 < pH ≤ 7.5 | 1.62 | Hg ≤ 4 | Low THg |
| u39 | Upland | 7.24 | 6.5 < pH ≤ 7.5 | 1.68 | Hg ≤ 4 | Low THg |
| u40 | Upland | 6.92 | 6.5 < pH ≤ 7.5 | 0.55 | Hg ≤ 4 | Low THg |
| u41 | Upland | 6.74 | 6.5 < pH ≤ 7.5 | 0.50 | Hg ≤ 4 | Low THg |
| u42 | Upland | 6.59 | 6.5 < pH ≤ 7.5 | 0.50 | Hg ≤ 4 | Low THg |
| u43 | Upland | 5.09 | pH ≤ 5.5 | 1.27 | Hg ≤ 2 | Low THg |
| u44 | Upland | 4.92 | pH ≤ 5.5 | 1.28 | Hg ≤ 2 | Low THg |
| u45 | Upland | 5.01 | pH ≤ 5.5 | 1.26 | Hg ≤ 2 | Low THg |
| u46 | Upland | 6.66 | 6.5 < pH ≤ 7.5 | 1.38 | Hg ≤ 4 | Low THg |
| u47 | Upland | 6.67 | 6.5 < pH ≤ 7.5 | 1.47 | Hg ≤ 4 | Low THg |
| u48 | Upland | 6.73 | 6.5 < pH ≤ 7.5 | 1.47 | Hg ≤ 4 | Low THg |
| u52 | Upland | 6.75 | 6.5 < pH ≤ 7.5 | 2.72 | Hg ≤ 4 | Low THg |
| u53 | Upland | 6.62 | 6.5 < pH ≤ 7.5 | 2.71 | Hg ≤ 4 | Low THg |
| u54 | Upland | 6.76 | 6.5 < pH ≤ 7.5 | 2.97 | Hg ≤ 4 | Low THg |
| u58 | Upland | 8.07 | pH > 7.5 | 1.12 | Hg ≤ 6 | Low THg |
| u59 | Upland | 7.65 | pH > 7.5 | 1.06 | Hg ≤ 6 | Low THg |
| u60 | Upland | 7.39 | 6.5 < pH ≤ 7.5 | 1.12 | Hg ≤ 4 | Low THg |
| u61 | Upland | 7.2 | 6.5 < pH ≤ 7.5 | 1.13 | Hg ≤ 4 | Low THg |
| u62 | Upland | 7.15 | 6.5 < pH ≤ 7.5 | 1.10 | Hg ≤ 4 | Low THg |
| u63 | Upland | 7.17 | 6.5 < pH ≤ 7.5 | 1.12 | Hg ≤ 4 | Low THg |
| u64 | Upland | 7.07 | 6.5 < pH ≤ 7.5 | 1.10 | Hg ≤ 4 | Low THg |
| u65 | Upland | 7.07 | 6.5 < pH ≤ 7.5 | 1.25 | Hg ≤ 4 | Low THg |
| u66 | Upland | 7.07 | 6.5 < pH ≤ 7.5 | 1.17 | Hg ≤ 4 | Low THg |
| u67 | Upland | 6.91 | 6.5 < pH ≤ 7.5 | 1.41 | Hg ≤ 4 | Low THg |
| u68 | Upland | 7.02 | 6.5 < pH ≤ 7.5 | 1.42 | Hg ≤ 4 | Low THg |
| u69 | Upland | 7.1 | 6.5 < pH ≤ 7.5 | 1.42 | Hg ≤ 4 | Low THg |
| p4 | Paddy | 6.91 | 6.5 < pH ≤ 7.5 | 39.48 | Hg ≤ 4 | High THg |
| p5 | Paddy | 6.8 | 6.5 < pH ≤ 7.5 | 38.02 | Hg ≤ 4 | High THg |
| p6 | Paddy | 6.71 | 6.5 < pH ≤ 7.5 | 38.02 | Hg ≤ 4 | High THg |
| p16 | Paddy | 7.31 | 6.5 < pH ≤ 7.5 | 26.03 | Hg ≤ 4 | High THg |
| p17 | Paddy | 7.23 | 6.5 < pH ≤ 7.5 | 23.41 | Hg ≤ 4 | High THg |
| p18 | Paddy | 7.19 | 6.5 < pH ≤ 7.5 | 24.03 | Hg ≤ 4 | High THg |
| p22 | Paddy | 7.45 | 6.5 < pH ≤ 7.5 | 52.37 | Hg ≤ 4 | High THg |
| p23 | Paddy | 7.21 | 6.5 < pH ≤ 7.5 | 52.34 | Hg ≤ 4 | High THg |
| p24 | Paddy | 7.3 | 6.5 < pH ≤ 7.5 | 44.99 | Hg ≤ 4 | High THg |
| p25 | Paddy | 6.88 | 6.5 < pH ≤ 7.5 | 30.41 | Hg ≤ 4 | High THg |
| p26 | Paddy | 6.82 | 6.5 < pH ≤ 7.5 | 29.86 | Hg ≤ 4 | High THg |
| p27 | Paddy | 6.7 | 6.5 < pH ≤ 7.5 | 30.00 | Hg ≤ 4 | High THg |
| p28 | Paddy | 6.17 | 5.5 < pH ≤ 6.5 | 4.78 | Hg ≤ 2.5 | High THg |
| p29 | Paddy | 5.93 | 5.5 < pH ≤ 6.5 | 3.88 | Hg ≤ 2.5 | High THg |
| p30 | Paddy | 5.83 | 5.5 < pH ≤ 6.5 | 4.22 | Hg ≤ 2.5 | High THg |
| p31 | Paddy | 6.82 | 6.5 < pH ≤ 7.5 | 22.41 | Hg ≤ 4 | High THg |
| p32 | Paddy | 6.84 | 6.5 < pH ≤ 7.5 | 26.90 | Hg ≤ 4 | High THg |
| p33 | Paddy | 6.87 | 6.5 < pH ≤ 7.5 | 28.83 | Hg ≤ 4 | High THg |
| p49 | Paddy | 6.18 | 5.5 < pH ≤ 6.5 | 3.53 | Hg ≤ 2.5 | High THg |
| p50 | Paddy | 6.31 | 5.5 < pH ≤ 6.5 | 3.63 | Hg ≤ 2.5 | High THg |
| p51 | Paddy | 6.14 | 5.5 < pH ≤ 6.5 | 3.68 | Hg ≤ 2.5 | High THg |
| p52 | Paddy | 7.14 | 6.5 < pH ≤ 7.5 | 5.55 | Hg ≤ 4 | High THg |
| p53 | Paddy | 7.21 | 6.5 < pH ≤ 7.5 | 5.22 | Hg ≤ 4 | High THg |
| p54 | Paddy | 6.94 | 6.5 < pH ≤ 7.5 | 5.86 | Hg ≤ 4 | High THg |
| p56 | Paddy | 7.38 | 6.5 < pH ≤ 7.5 | 4.16 | Hg ≤ 4 | High THg |
| p58 | Paddy | 7.11 | 6.5 < pH ≤ 7.5 | 6.84 | Hg ≤ 4 | High THg |
| p59 | Paddy | 6.96 | 6.5 < pH ≤ 7.5 | 7.25 | Hg ≤ 4 | High THg |
| p60 | Paddy | 6.77 | 6.5 < pH ≤ 7.5 | 7.72 | Hg ≤ 4 | High THg |
| u17 | Upland | 5.99 | 5.5 < pH ≤ 6.5 | 2.79 | Hg ≤ 2.5 | High THg |
| u19 | Upland | 6.99 | 6.5 < pH ≤ 7.5 | 16.44 | Hg ≤ 4 | High THg |
| u20 | Upland | 6.99 | 6.5 < pH ≤ 7.5 | 15.72 | Hg ≤ 4 | High THg |
| u21 | Upland | 7.04 | 6.5 < pH ≤ 7.5 | 15.32 | Hg ≤ 4 | High THg |
| u22 | Upland | 7.12 | 6.5 < pH ≤ 7.5 | 19.14 | Hg ≤ 4 | High THg |
| u23 | Upland | 7.19 | 6.5 < pH ≤ 7.5 | 17.95 | Hg ≤ 4 | High THg |
| u24 | Upland | 7.2 | 6.5 < pH ≤ 7.5 | 19.66 | Hg ≤ 4 | High THg |
| u28 | Upland | 7.22 | 6.5 < pH ≤ 7.5 | 7.77 | Hg ≤ 4 | High THg |
| u29 | Upland | 7.39 | 6.5 < pH ≤ 7.5 | 7.97 | Hg ≤ 4 | High THg |
| u30 | Upland | 7.34 | 6.5 < pH ≤ 7.5 | 7.90 | Hg ≤ 4 | High THg |
| u31 | Upland | 7.57 | pH > 7.5 | 32.43 | Hg ≤ 6 | High THg |
| u32 | Upland | 7.7 | pH > 7.5 | 39.54 | Hg ≤ 6 | High THg |
| u33 | Upland | 7.4 | 6.5 < pH ≤ 7.5 | 34.85 | Hg ≤ 4 | High THg |
| u49 | Upland | 7.48 | 6.5 < pH ≤ 7.5 | 11.63 | Hg ≤ 4 | High THg |
| u50 | Upland | 7.63 | pH > 7.5 | 11.48 | Hg ≤ 6 | High THg |
| u51 | Upland | 7.68 | pH > 7.5 | 10.74 | Hg ≤ 6 | High THg |
| u55 | Upland | 6.44 | 5.5 < pH ≤ 6.5 | 6.81 | Hg ≤ 2.5 | High THg |
| u56 | Upland | 6.6 | 6.5 < pH ≤ 7.5 | 6.91 | Hg ≤ 4 | High THg |
| u57 | Upland | 6.75 | 6.5 < pH ≤ 7.5 | 6.79 | Hg ≤ 4 | High THg |
